# RNF8 mediates NONO degradation following UV-induced DNA damage to properly terminate ATR-CHK1 checkpoint signaling

**DOI:** 10.1093/nar/gky1166

**Published:** 2018-11-16

**Authors:** Rakesh Deshar, Wonjin Yoo, Eun-Bee Cho, Sungjoo Kim, Jong-Bok Yoon

**Affiliations:** 1 Department of Medical Lifesciences, The Catholic University of Korea, Seoul 137-701, Korea; 2 Department of Biochemistry, College of Life Science & Biotechnology, Yonsei University, Seoul 120-749, Korea

## Abstract

RNF8 plays a critical role in DNA damage response (DDR) to initiate ubiquitination-dependent signaling. To better characterize the role of RNF8 in UV-induced DDR, we searched for novel substrates of RNF8 and identified NONO as one intriguing substrate. We found that: (i) RNF8 ubiquitinates NONO and (ii) UV radiation triggers NONO ubiquitination and its subsequent degradation. Depletion of RNF8 inhibited UV-induced degradation of NONO, suggesting that RNF8 targets NONO for degradation in response to UV damage. In addition, we found that 3 NONO lysine residues (positions 279, 290 and 295) are important for conferring its instability in UV-DDR. Depletion of RNF8 or expression of NONO with lysine to arginine substitutions at positions 279, 290 and 295 prolonged CHK1 phosphorylation over an extended period of time. Furthermore, expression of the stable mutant, but not wild-type NONO, induced a prolonged S phase following UV exposure. Stable cell lines expressing the stable NONO mutant showed increased UV sensitivity in a clonogenic survival assay. Since RNF8 recruitment to the UV-damaged sites is dependent on ATR, we propose that RNF8-mediated NONO degradation and subsequent inhibition of NONO-dependent chromatin loading of TOPBP1, a key activator of ATR, function as a negative feedback loop critical for turning off ATR-CHK1 checkpoint signaling in UV-DDR.

## INTRODUCTION

DNA damage elicits a network of cellular pathways termed DNA damage response (DDR) to: (i) activate cell cycle checkpoints and repair the damaged DNA, or (ii) induce apoptosis when DNA injury is severe and irreparable (1–3). Post-translational modifications (PTMs), including ubiquitination, play key roles in coordinating DDR (4). RING finger protein 8 (RNF8) is a major E3 ubiquitin ligase that rapidly accumulates at sites of DNA damage through its FHA domain-mediated interaction with phosphorylated MDC1; MDC1 is phosphorylated in response to DNA damage by phosphoinositol-3-kinase-related kinases, such as Ataxia telangiectasia mutated (ATM) and ATM and Rad3-related (ATR) (5–8). The lysine 63-linked polyubiquitination of H1-type linker histones by RNF8 recruits the downstream E3 ligase RNF168 to further amplify the ubiquitination of H2A and H2AX histones (9,10). Through this signal amplification step, a number of repair proteins such as p53-binding protein 1 (53BP1) and breast cancer susceptibility protein 1 (BRCA1) are recruited to the damaged chromatin (11,12). In addition to synthesizing lysine 63-linked polyubiquitin chains, RNF8 also mediates the lysine 48-linked polyubiquitination and degradation of DDR proteins including KU80 (13), checkpoint kinase 2 (CHK2) (13), 53BP1 (14), the lysine demethylase KDM4A (JMJD2A) (15), and the p12 subunit of DNA polymerase δ (16) to modulate their function in DDR.

Non-POU domain-containing octamer-binding protein (NONO) is a multi-functional nuclear protein which binds both RNA and DNA (17,18). NONO belongs to the Drosophila behavior/human splicing (DBHS) protein family (19), which, in humans, contains two additional members, splicing factor proline/glutamine-rich (SFPQ) and paraspeckle protein component 1 (PSPC1). DBHS members form stable dimers with each other and function in various aspects of RNA processing and gene expression (19,20). NONO is involved in transcriptional regulation (21–23), mRNA splicing and processing (24–26), nuclear retention of inosine-containing RNAs (27), circadian clock (28,29), and paraspeckle formation (30). Recent studies (31–39) link NONO and its binding partner SFPQ to double-strand break (DSB)-induced DDR and DNA repair by nonhomologous end joining (NHEJ) and homologous recombination. Detailed in vitro analysis with the purified heterodimer of NONO and SFPQ showed that it stimulates DNA ligase IV and XRCC4-directed end joining by promoting DNA substrate pairing (40–42). Consistent with its role in DNA repair, NONO is transiently recruited to DNA damage sites (32,35,43) through its interaction with poly (ADP-ribose) (35) and its retention at the damage sites is affected by its interacting protein Matrin 3 (43). Apart from its role in DSB repair, NONO is involved in UV-induced DDR. It was recently reported that NONO plays an important role in triggering the intra-S-phase checkpoint through activation of ATR-CHK1 signaling cascade in response to UV-induced DNA damage (44).

Since RNF8 is a key E3 ubiquitin ligase functioning in both DSB- and UV-induced DDR, identification of additional substrates will help further elucidate its role in DNA damage signaling. To identify substrates of ubiquitin ligases, we have recently devised a method based on proximity-dependent biotin labeling (45,46). In this method, an E3 ubiquitin ligase of interest is expressed as a fusion to *Escherichia coli* biotin ligase BirA together with a biotin acceptor peptide (AP)-tagged ubiquitin. The BirA-directed biotin labeling of AP depends on the proximity of the two fusion proteins in the cell, which leads to preferential labeling of ubiquitinated E3 substrates. In this study, we applied this procedure to RNF8 and identified NONO as an intriguing substrate. We found that UV-induced NONO degradation by RNF8 is required for timely termination of intra-S-phase checkpoint signaling and continued cell cycle progression.

## MATERIALS AND METHODS

### Plasmid constructs

NONO, RNF8, RNF168 and Rad18 cDNAs were purchased from Korean Human Gene Bank, Medical Genomics Research Center, KRIBB, Korea. The cDNAs were PCR amplified with gene-specific primer sets (NONO: 5′-GGATCCATGCAGAGTAATAAAACTTTTAAC-3′ [sense] and 5′-CTCGAGTTAGTATCGGCGACGTTTGTT-3′ [antisense]; RNF8: 5′-AGATCTATGGAGCCCGGCTTCTTC-3′ [sense] and 5′-CTCGAGTCAGAACAATCTCTTTGCTTTTCG-3′ [antisense]; RNF168: 5′-AGATCTATGGCTCTACCCAAAGAC-3′ [sense] and 5′-CTCGAGTTACTTTGTGCATCTCTG-3′ [antisense]; Rad18: 5′-GGATCCATGGACTCCCTGGCCGAG-3′ [sense] and 5′-CTCGAGTTAATTCCTATTACGCTTGTTTCT-3′ [antisense]; PSPC1: 5′- CGCGGATCCATGATGTTAAGAGGAAACCTG-3′ [sense] and 5′-CCGCTCGAGTTAATATCTACGACGCTTATTAG-3′ [antisense]; SFPQ: 5′- CCGCTCGAGCATGTCTCGGGATCGGTTCC-3′ [sense] and 5′- CTAGCTAGCCTAAAATCGGGGTTTTTTGTTTG-3′ [antisense]) and subcloned into either the pCDNA 3.1 (Invitrogen) or the pEGFP-C1 (CLONTECH). Sequences required for generating NONO-deletion mutants (NONO 1-309, NONO 1-276, NONO 1-226, NONO 60-471, NONO 180-471 and NONO 277-471) were amplified by PCR with mutation-specific primer sets (NONO 1-309: 5′-GCGGCCGCAATGCAGAGTAATAAAACTTTTAAC-3′ [sense] and 5′-GCGGCCGCGACCTGGTGCTCATGGCGT-3′ [antisense]; NONO 1-276: 5′-GCGGCCGCAATGCAGAGTAATAAAACTTTTAAC-3′ [sense] and 5′-GCGGCCGCCTCAATGAGTGCCTTCCAG-3′ [antisense]; NONO 1-226: 5′- GCGGCCGCAATGCAGAGTAATAAAACTTTTAAC-3′ [sense] and 5′- GCGGCCGCGGGCTCCACAGTCACAGG-3′ [antisense]; NONO 60-471: 5′-GCGGCCGCAATGAAGAATTTTAGAAAACCAGG-3′ [sense] and 5′-GCGGCCGCGTATCGGCGACGTTTGTT-3′ [antisense]; NONO 180-471 5′-GCGGCCGCAATGATTGTGGATGATCGAGGAAG-3′ [sense] and 5′-GCGGCCGCGTATCGGCGACGTTTGTT-3′ [antisense]; NONO 277-471: sense primer 5′-GCGGCCGCAATGGAGAAGCAGCAGCAG-3′, and 5′-GCGGCCGCGTATCGGCGACGTTTGTT-3′ [antisense]) and inserted into the pEF/myc/nuc/GFP vector (Invitrogen). To generate the NONO Δ277-308 mutant construct, two specific fragments of NONO (designated A and B) were amplified using PCR with unique primer sets (fragment A: 5′-GGATCCATGCAGAGTAATAAAACTTTTAAC-3′ [sense] and 5′GAATTCCTCAATGAGTGCCTTCCAG-3′ [antisense]; fragment B: 5′-GAATTCATGCTAATGAGACAGGATTT-3′ [sense] and 5′-CTCGAGTTAGTATCGGCGACGTTTGTT-3′ [antisense]) were ligated and inserted into the pCDNA 3.1 vector. Site-directed mutagenesis was performed using a QuikChange Kit according to the manufacturer's protocol (Stratagene) with the following primer sets: RNF8 C403S: 5′-AGAGAATGAGCTCCAAAGTATTATTTGTTCAGAATA-3′ and 5′-TATTCTGAACAAATAATACTTTGGAGCTCATTCTCT-3′; NONO K279R: 5′-TCATTGAGATGGAGAGGCAGCAGCAGGACCA-3′ and 5′-TGGTCCTGCTGCTGCCTCTCCATCTCAATGA-3′; NONO K290R: 5′-GGACCGCAACATCAGGGAGGCTCGTGAGA-3′ and 5′-TCTCACGAGCCTCCCTGATGTTGCGGTCC-3′; NONO K295R: 5′-GGAGGCTCGTGAGAGGCTGGAGATGGAGA-3′ and 5′-TCTCCATCTCCAGCCTCTCACGAGCCTCC-3′.

### Cell culture, transfection and reagents

HeLa, U2OS, CHO, A549 and HEK-293 cells were maintained in DMEM (WelGENE) supplemented with 10% fetal bovine serum (GIBCO), 100 units/ml penicillin, and 100 μg/ml streptomycin at 37°C, 5% CO_2_. Cells were transiently transfected using polyethylenimine (Sigma) or Lipofectamine 2000 reagent (Invitrogen). Cytosine B-D-arabinofuranoside (AraC), hydroxyurea (HU) and cycloheximide (CHX) were obtained from Sigma. MG132 was obtained from Cayman Chemical Company. Propidium iodide was purchased from Roche Diagnostics.

### RNA interference

NONO siRNA duplexes (5′-CAGGCGAAGUCUUCAUUCA-3′) (47), RNF8 siRNA duplexes (5′-UGCGGAGUAUGAAUAUGAA-3′) (48,49), XPC siRNA duplexes (5′-GGA GGGCGAUGAAACGUUU-3′) (50) and control siRNA duplexes (5′-CCUACGCCACCAAUUUGGU-3′) were synthesized at Bioneer. siRNA transfection was performed using Lipofectamine RNAimax reagent (Invitrogen) according to the manufacturer's instructions.

### Western blot analysis

Western blot and detection were performed as previously described (45). The following antibodies were used for Western blotting: anti-RNF8 (B-2) (#sc-271462, 1:5000), anti-GFP (#sc-9996, 1:3000), anti-Ub (A-5) (#sc-166553, 1:1000), anti-CDC25A (F-6) (#sc-7389, 1:1000), anti-p53 (#sc-126, 1:1000) and anti-HSP90 (H-114) (#sc-7479, 1:2000) from Santa Cruz Biotechnology; anti-phospho H2AX S139 (#ab11174, 1:20000), anti-H2AX (#ab11175, 1:10 000) and anti-TOPBP1 (#ab2402, 1:5000) from Abcam; anti-phospho CHK1 S345 (#2348, 1:2500), anti-CHK1 (#sc-8408, 1:3000) and anti-MYC (#2276, 1:2000) from Cell Signaling Technology; anti-FLAG (#F3165, 1:10 000), anti-biotin (#B7653, 1:10 000) and anti-β-actin (#A2228, 1:10000) from Sigma; anti-NONO (#611279, 1:10000) from BD Transduction Laboratories; anti-T7 (#69522, 1:10000) from Novagen; anti-HA (#clone 12CA5, 1:1000) from Roche; anti-ATRIP (#A300-095A, 1:2000), anti-XPC (#A301-122A, 1:2000) and anti-SFPQ (#A301-320A, 1:2000) from Bethyl Laboratories, Inc.; anti-K48-specific ubiquitin (#clone Apu2.07, 1:1000) and anti-K63-specific ubiquitin (#clone Apu3.A8, 1:1000) from Genentech Inc.

### Affinity purification of biotinylated proteins

The biotinylated-ubiquitinated proteins were purified from HeLa cells transfected with constructs encoding FLAG-BirA-RNF8 and AP-HA-Ub using previously described methods (45).

### Protein digestion and affinity purification of ubiquitinated peptides

The biotinylated-ubiquitinated proteins immobilized on beads were reduced, alkylated, trypsinized, and then purified by Ubiquitin Branch Motif (K-ϵ-GG) affinity purification procedure as described previously (45). For the identification of ubiquitination sites on NONO, HeLa cells were transfected with constructs encoding FLAG-NONO and HA-RNF8. 24 h post-transfection, cells were lysed in 1% (w/v) SDS (PBS-SDS) and clarified at 10,000 × *g* at 4°C for 10 min. The cell lysates were diluted 10-fold in 0.5% NP-40 lysis buffer (50 mM Tris-Cl, pH 7.4, 150 mM NaCl, 0.5% NP-40 and 0.2 mM PMSF) and immunoprecipitated with anti-FLAG M2 agarose overnight at 4°C. The beads were sequentially washed with 0.5% NP-40 lysis buffer, Tris-buffered saline (50 mM NaCl, 50 mM Tris–Cl, pH 7.4) and 100 mM ammonium bicarbonate (pH 7.8), and resuspended in 100 mM ammonium bicarbonate (pH 7.8). The immunoprecipitated ubiquitinated NONO proteins were reduced, alkylated, trypsinized, and then purified as described above.

### Mass spectrometry

The purified peptides were analyzed and quantitated by nanoelectrospray LC–MS/MS on an LTQ Orbitrap Velos (Thermo Fisher Scientific Inc.) as described previously (45).

### Expression and purification of recombinant proteins

His6-tagged human UbcH5a and human Ub were expressed in *Escherichia coli* strain BL21 (DE3) and purified using Ni2+-agarose beads (Qiagen). FLAG-tagged Uba1, FLAG-tagged RNF8 and HA-tagged NONO were expressed in HeLa cells and purified with FLAG-M2-agarose columns (Sigma) or HA-M2-agarose columns (Sigma). After extensively washing the column with high salt wash buffer (50 mM Tris–Cl, pH 7.4, 400 mM NaCl, 0.5% NP-40, 10 mM NaF, 1 mM Na_3_VO_4_ and 0.2 mM PMSF) to remove all non-covalently associated proteins, the bound proteins were eluted with lysis buffer containing 0.3 mg/ml FLAG peptide (Sigma) or 0.3 mg/ml HA peptide (Sigma).

### *In vitro* ubiquitination assay

The ubiquitination assay was performed in a 20 μl reaction volume containing 25 mM Tris–Cl, pH 7.4, 50 mM NaCl, 5 mM ATP, 10 mM MgCl_2_, 1 mM DTT, 800 ng of ubiquitin, 100 ng of Uba1, 600 ng of UbcH5a, 300 ng of RNF8, and 300 ng of NONO. Reaction mixtures were incubated at 37°C for 1 h, terminated by adding 2× Laemmli sample buffer, resolved by SDS–PAGE followed by western blot analysis.

### *In vivo* ubiquitination assay

HeLa cells were irradiated with UV (30 J/m^2^) for the designated time periods. Following UV irradiation, cells were harvested with ice-cold PBS and lysed in lysis buffer containing 1% (w/v) SDS (PBS-SDS), and 10 mM *N*-ethylmaleimide. The cell lysates were incubated at 90°C for 10 min to dissociate proteins followed by clarification at 10 000 × *g* at 4°C for 10 min. The lysate was then diluted 1:10 with 0.5% NP-40 lysis buffer (50 mM Tris–Cl, pH 7.4, 150 mM NaCl, 0.5% NP-40 and 0.2 mM PMSF). For immunoprecipitation of endogenous NONO, lysates were incubated with 1 μg of anti-NONO antibody and Protein G-agarose beads (Invitrogen) overnight at 4°C with rotation. For immunoprecipitation of FLAG-tagged or HA-tagged protein, lysates were incubated with anti-FLAG-M2-agarose or anti-HA-M2-agarose overnight at 4°C. After beads were washed three times with 0.5% NP-40 lysis buffer, samples were eluted with 2× Laemmli sample buffer and analyzed by SDS PAGE and western blotting.

### UV irradiation and UV clonogenic survival assay

Cells were washed once with PBS and globally exposed to 30 J/m^2^ UV radiation (UV-C, 254 nm) using CL-1000 UV Crosslinker (UVP, Inc.). After exposure, the cells were cultured for the indicated time periods. For local UV irradiation, cells were rinsed once with PBS and were masked with 5 μm filter (#TMTP02500, Merck Millipore Corporation) and irradiated with UV (100 J/m^2^) (51). AraC and HU at final concentrations of 10 μM and 50 mM respectively, were added to the medium 3 h before irradiation and remained throughout the time course of the experiment. UV clonogenic survival assay was performed as previously described (52).

### Immunofluorescence microscopy

Immunofluorescence assays were performed as previously reported (45). The following antibodies were used for immunofluorescence staining: anti-NONO (#611279, 1:500) from BD Transduction Laboratories; anti-SFPQ (#A301-320A, 1:200) from Bethyl Laboratories, Inc.; anti-phospho H2AX S139 (#ab11174, 1:1000) from Abcam.

### Chromatin fractionation

Chromatin fractionation was performed as described previously (53–55) with minor modifications. Briefly, HeLa cells were harvested and washed with PBS. Cells were resuspended in Buffer A (10 mM HEPES, pH 8.0, 10 mM KCl, 1.5 mM MgCl_2_, 1 mM EDTA, 10% glycerol, 1 mM DTT, 0.5% Triton-X-100, 10 mM NaF, 1 mM Na_3_VO_4_ and 0.2 mM PMSF) and kept on ice for 8 min. After centrifugation at 1300 × *g* for 5 min at 4°C, the cell lysates were separated into soluble and nuclei fractions. The soluble fraction was further clarified by centrifugation at 12 000 × *g* for 15 min. To obtain chromatin fractions, the obtained nuclei pellet was washed with Buffer A at 1300 × *g* for 4 min at 4°C and resuspended in Laemmli sample buffer.

### FACS analysis

Flow cytometry analysis was performed using propidium iodide staining. Following UV exposure, cells were recovered in complete medium in a 37°C incubator for the designated time intervals. Cells were harvested by trypsinization and washed with PBS. After resuspension in 0.3 ml of PBS, cells were fixed with 0.7 ml of 100% ethanol and kept at 4°C overnight. Cells were then washed with 1% (w/v) BSA (PBS-BSA) and resuspended in 0.5 ml of PBS. 100 μg/ml of RNase (Sigma) was added to the cell suspension and incubated at 37°C for 1 h. Cells were stained with 30 μg/ml propidium iodine (Sigma) in the absence of light and at room temperature for 1 h. The cell cycle profiles were analyzed by flow cytometric analysis with FACScalibur (BD Biosciences) using Cell Quest and were modeled by used of the ModFit LT software (Version 4.1, Verity Software House, Inc.).

### Comet assay

Neutral comet assay was performed as previously described (56). Briefly, cell suspension of control siRNA- or XPC siRNA-treated cells was mixed in low-melting agarose and add onto the slides. After agarose gel solidified, slides were incubated with neutral lysis buffer (2% sodium lauryl sarcosinate, 0.5 M Na_2_EDTA pH 8.0) at 4°C for overnight in the dark and run at 0.6 V/cm for 30 min. Nuclei were stained with 2.5 μg/ml propidium iodide, visualized under fluorescence microscopy, and analyzed by using CometScore version 2.0 software (TriTek Corporation, Sumerduck, VA, USA).

## RESULTS

### RNF8 promotes ubiquitination and subsequent degradation of NONO

To identify potential substrates of RNF8, cells were transfected with FLAG-BirA-RNF8 and AP-HA-Ub expression constructs and labeled with biotin for 1 h. Since BirA can biotinylate AP only when they make direct contact with each other in the presence of biotin, both AP-HA-Ub bound on E2 and AP-HA-Ub conjugates on substrates will be preferentially biotin-labeled by FLAG-BirA-RNF8 during the ubiquitination reaction due to their close proximity. Biotin-labeled proteins were purified using streptavidin beads and digested with trypsin for subsequent enrichment of diGly peptides with diGly antibody-conjugated beads. Mass spectrometric analysis of the diGly peptide preparation identified peptides from a group of potential substrates of RNF8 including NONO (Supplementary Figure S1) [the data has been deposited at ProteomeXchange database, www.proteomexchange.org, identifier: PXD006156]. To directly examine whether NONO is biotin labeled by BirA-RNF8 and AP-Ub, cells were transfected with FLAG-BirA-RNF8, HA-NONO and AP-Ub, and treated with biotin. HA-NONO was first immunoprecipitated with an anti-HA antibody and immunoblotted using an anti-biotin antibody. Strong biotin labeling of HA-NONO was observed with FLAG-BirA-RNF8, but not with FLAG-BirA or FLAG-BirA-RNF8 C403S, an RNF8 mutant deficient in E3 ligase activity (57,58) (Supplementary Figure S2), suggesting that NONO is a substrate of RNF8.

**Figure 1.**
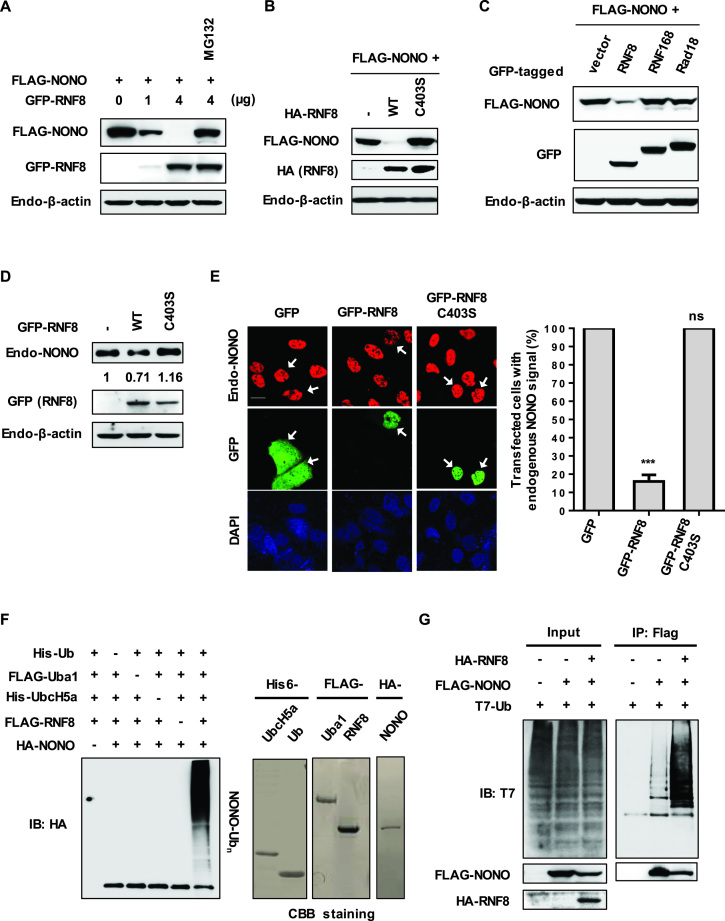
RNF8 promotes the ubiquitination and subsequent degradation of NONO. (**A**) Proteasome inhibitor MG132 blocks RNF8-mediated NONO degradation. HeLa cells were transfected with plasmid encoding FLAG-NONO, along with increasing concentrations of GFP-RNF8 expression plasmid (from 1–4 μg) and treated with DMSO or 25 μM MG132 for 4 h. Cell lysates were then immunoblotted with the indicated antibodies. (**B**) RNF8 E3 ligase activity is required for the degradation of NONO. HeLa cells co-transfected with FLAG-NONO plus an empty vector or HA-RNF8 or HA-RNF8 C403S were harvested and processed for Western blotting analysis. (**C**) RNF8, but not RNF168 or Rad18, mediates the degradation of NONO. HeLa cells were co-transfected with FLAG-NONO plus an empty vector or GFP-RNF8 or GFP-RNF168 or GFP-Rad18 and their whole cell lysates were immunoblotted with the indicated antibodies. (**D**) Exogenously expressed RNF8 degrades endogenous NONO. Cells were transiently transfected with GFP-RNF8 or GFP-RNF8 C403S. Cell lysates were probed with the indicated antibodies for Western blotting analysis. (**E**) HeLa cells were transfected with plasmid encoding GFP, GFP-RNF8 or GFP-RNF8 C403S. The cells were immunostained with the indicated antibodies and visualized using confocal microscopy. Arrows indicate cells expressing GFP, GFP-RNF8 or GFP-RNF8 C403S. Scale bar, 20 μm. Quantification of endogenous NONO signal in transfected cells was presented in the histogram (right panel). Data shown are representative of at least three independent experiments containing more than 100 cells/sample. Error bars, SD. ***P < 0.001 Student's *t*-test; n.s. not significant. (**F**) RNF8 ubiquitinates NONO *in vitro*. For the *in vitro* ubiquitination assay, reaction mixtures of Uba1 (E1), UbcH5a (E2), RNF8 (E3), ubiquitin, an ATP-generating system and HA-NONO (substrate) were used. Reaction mixtures were incubated for 1 h at 37°C. The products were pulled down with HA beads and analyzed by SDS-PAGE followed by Western blotting for ubiquitin (left panel). The purified proteins His6-UbcH5a, His6-Ub, FLAG-Uba1, FLAG-RNF8 and HA-NONO used in *in vitro* ubiquitination assay were visualized by Coomassie Brilliant Blue (CBB) staining (right panel). (**G**) RNF8 catalyzes *in vivo* NONO ubiquitination. HeLa cells were transfected with T7-Ub alone or in combination with T7-Ub and FLAG-NONO or T7-Ub, FLAG-NONO and HA-RNF8. Cells were lysed and processed for *in vivo* ubiquitination assay. Immunoprecipitates with an anti-FLAG antibody were probed with antibodies as indicated.

To confirm this further, we examined whether RNF8 overexpression can affect NONO expression by transfecting HeLa cells with expression vectors of GFP-RNF8 and FLAG-NONO. Levels of FLAG-NONO were dramatically reduced in cells transfected with GFP-RNF8 in a concentration-dependent manner (Figure 1A). The decrease of FLAG-NONO was blocked following treatment with the proteasome inhibitor MG132, indicating that degradation of FLAG-NONO is proteasome dependent (lane 4 in Figure 1A). In contrast to the wild-type ligase, HA-RNF8 C403S failed to reduce FLAG-NONO levels (Figure 1B), showing that the E3 ligase activity is required for RNF8-mediated NONO degradation. We also observed that MG132 treatment has no significant effect on the ectopically expressed NONO protein level without RNF8 overexpression (Supplementary Figure S3). Downregulation of NONO appears to be specific for RNF8, since levels of FLAG-NONO were not affected by RNF168 and Rad18, E3 ligases known to be linked to DDR (Figure 1C).

Next, we tested whether endogenous NONO is affected by RNF8 overexpression. The level of endogenous NONO is decreased in cells transfected with GFP-tagged RNF8 wild-type, but not GFP-RNF8 C403S (Figure 1D). The results of fluorescence confocal microscopy of these transfected cells further confirms that endogenous NONO is lowered only in cells transfected with wild-type RNF8 (Figure 1E). Remarkably, endogenous SFPQ, another member of DBHS family, was not reduced by overexpression of RNF8 (Supplementary Figure S4), showing the substrate specificity of RNF8 towards NONO.

To determine if RNF8 directs NONO ubiquitination, we performed an *in vitro* ubiquitination assay using purified components. Efficient ubiquitination of NONO was observed in the presence of E1, E2, E3 and Ub (Figure 1F). Deleting any of the components failed to promote ubiquitination of NONO. To further test if RNF8 ubiquitinates NONO *in vivo*, we transfected HeLa cells with expression constructs for HA-RNF8, FLAG-NONO and T7-Ub. FLAG-NONO was immunoprecipitated with an anti-FLAG antibody and tested for the conjugation of T7-Ub by immunoblotting with an anti-T7 antibody. Expression of RNF8 generated a ubiquitination smear of FLAG-NONO (Figure 1G). In addition, we found that RNF8 mediates both K63- and K48-linked polyubiquitination on NONO as shown in Supplementary Figure S5A and B. Taken together, these results indicate that RNF8 ubiquitinates NONO and results in subsequent proteasome-mediated degradation.

### DNA damage induced by UV leads to degradation of NONO

**Figure 2.**
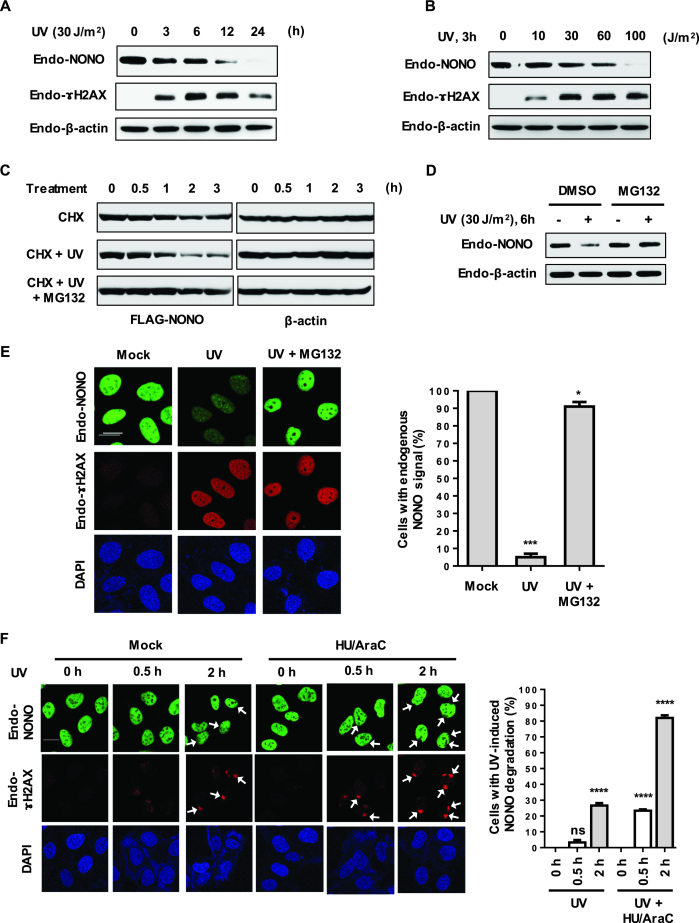
DNA damage induced by UV leads to degradation of NONO. (**A**) NONO is degraded after UV-induced DNA damage. HeLa cells were treated with a fixed dose of UV (30 J/m^2^) for the indicated time points. Cells were harvested and immunoblotted using indicated antibodies. (**B**) NONO degrades in a UV dose-dependent manner. HeLa cells were treated with increasing doses of UV for 3 h. Cell lysates were immunoblotted using indicated antibodies. (**C**) The proteasome inhibitor MG132 blocks UV-induced NONO degradation. HeLa cells transfected with FLAG-NONO were irradiated with UV (30 J/m^2^) in the presence of 50 μg/ml CHX with or without 25 μM MG132 for the indicated time points. Cell lysates were analyzed by Western blotting. (**D**) HeLa cells were treated with UV (30 J/m^2^) in the presence of DMSO or 25 μM MG132 for 6 h. The collected protein samples were immunoblotted with the indicated antibodies. (**E**) Immunofluorescence analysis of UV-induced NONO proteolysis occurring in a proteasome-dependent manner. U2OS cells were globally exposed to UV (30 J/m^2^) in the presence or absence of 10 μM MG132 for 12 h. Cells were fixed and treated with anti-NONO and anti-γH2AX antibodies for visualization. Scale bar, 20 μm. The histogram shows quantification of endogenous NONO signal detected from the experiments performed as in Figure 1E. Error bars, SD. *P < 0.05, ***P < 0.001 Student's *t*-test. (**F**) NER intermediates accelerate NONO degradation upon UV DNA damage. U2OS cells were locally exposed to UV (100 J/m^2^) with or without a pretreatment for 3 h with HU/Arac. After LUD for different periods of time, cells were fixed and immunostained with anti-NONO and anti-γH2AX antibodies. Arrows indicate LUD sites. Scale bar, 20 μm. The right panel shows quantification of endogenous NONO signal detected from the experiments performed as in Figure 1E. Error bars, SD. ****P < 0.0001 Student's *t*-test; n.s. not significant.

Since RNF8 plays a role in UV-induced DDR, we tested whether NONO levels were affected by UV radiation. UV irradiation of HeLa cells resulted in a gradual decrease in endogenous NONO levels as measured using Western blotting (Figure 2A). NONO was barely detectable at 12 h after exposure to 30 J/m^2^ UV radiation. Similar results were obtained using fluorescence confocal microscopy (Figure 2E). UV radiation reduced NONO in a dose-dependent manner (Figure 2B). Furthermore, we found that NONO is degraded in different human cell lines in response to UV radiation, suggesting that UV-induced NONO downregulation is well conserved process (Supplementary Figure S6).

To investigate whether UV-induced NONO reduction is caused by degradation of NONO, we blocked translation using cycloheximide and measured NONO stability following UV radiation. As shown in Figure 2C, UV radiation decreased the stability of NONO. Proteasome inhibition by MG132 blocked UV-mediated downregulation of NONO (Figure 2D and E). These results demonstrate that UV reduces NONO by inducing its degradation but not by blocking transcription of NONO by UV-generated kinks and bends of DNA at damage sites.

We next examined whether NONO was affected by ionizing radiation (IR), since RNF8 is known to play a role in repair of DSBs. We observed that NONO protein levels gradually decrease following IR (Supplementary Figure S7A). However, we found that there was little change in NONO ubiquitination following IR (Supplementary Figure S7B) and that the proteasome inhibitor was not able to block NONO reduction (Supplementary Figure S7C), indicating that IR-induced NONO reduction is not mediated by ubiquitin-dependent proteasome pathway.

Nucleotide excision repair (NER) plays a crucial role in the repair of UV-induced DNA damage. Previous studies have shown that NER-generated single-stranded DNA gaps activate ATR to phosphorylate MDC1, which in turn recruits RNF8 (8). Therefore, we sought to investigate whether the single-stranded NER intermediates influence UV-induced NONO degradation. For this, we utilized the DNA polymerase inhibitor AraC in combination with the ribonucleotide reductase inhibitor HU as this will inhibit gap filling and increase the amount of single-stranded NER intermediates. Following pretreatment with AraC/HU, U2OS cells were locally irradiated with UV and levels of NONO were monitored. Without the repair inhibition, we observed minimal NONO degradation at sites of local UV damage (LUD) 2 h following UV exposure (30 J/m^2^). In contrast, we obtained clear reduction of NONO at LUD sites in cells pretreated with AraC/HU (Figure 2F). To further support the notion that NER-dependent ATR activation is responsible for UV-induced NONO degradation, we depleted XPC, a critical DNA damage recognition protein involved in NER (59,60) using siRNA and examined NONO protein stability in response to UV radiation. We found that NONO degradation was dramatically inhibited in XPC depleted cells compared to control cells after UV exposure (Supplementary Figure S8). Collapse of stalled replication forks is known to generate DSBs during UV DNA damage (61). To examine whether UV-induced DSBs are involved in NONO degradation, we performed comet assay under neutral condition, which allows the detection of DNA double strand breaks only, in control siRNA or XPC siRNA-treated U2OS cells after UV irradiation. As expected, we observed the accumulation of DSBs after UV irradiation. However, there were no significant differences in the level of DSB accumulation in XPC depleted cells compared to control cells, suggesting that UV-induced DSBs have a minor effect on NONO degradation (Supplementary Figure S9). Overall, our findings suggest that UV radiation induces NONO degradation through NER-dependent ATR activation.

### UV-induced NONO degradation is mediated by RNF8

**Figure 3.**
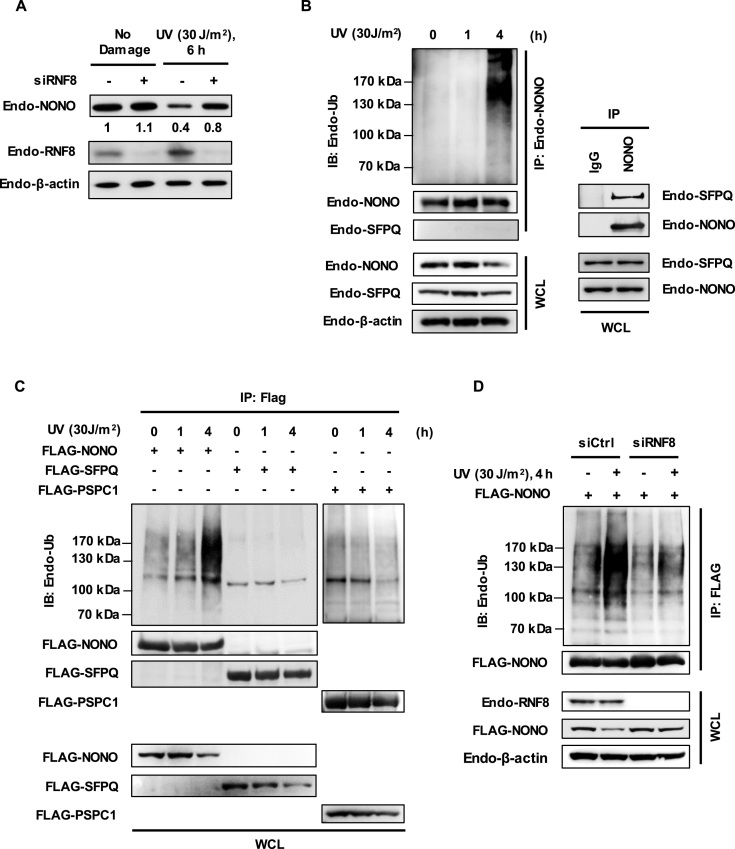
UV-induced NONO degradation is mediated by RNF8. (**A**) RNF8 depletion stabilizes NONO after UV damage. HeLa cells were transfected with control siRNA or RNF8 siRNA, and treated with UV (30 J/m^2^) for 6 h. Cell lysates were processed for immunoblotting with the indicated antibodies. (**B**) UV irradiation increases the polyubiquitination of NONO. HeLa cells were irradiated with UV (30 J/m^2^) for the indicated time points and processed for the *in vivo* ubiquitination assay (left panel). Immunoprecipitation experiment revealed that NONO interacted with SFPQ under the immunoprecipitation buffer condition containing 0.5% NP-40 (right panel). (**C**) UV-irradiation specifically ubiquitinates NONO but not SFPQ or PSPC1. *In vivo* ubiqutination assays of NONO, SFPQ and PSPC1 were performed with HeLa cells at the indicated time points following UV (30 J/m^2^) irradiation. (**D**) Knockdown of RNF8 impairs *in vivo* NONO ubiquitination after UV DNA damage. HeLa cells transfected with control siRNA or RNF8 siRNA were treated with UV (30 J/m^2^) for 4 h and processed for the *in vivo* ubiquitination assay. The ubiquitin-conjugated endogenous NONO was subsequently detected by Western blotting analysis using anti-Ub antibody.

Since RNF8 promotes NONO ubiquitination (Figure 1) and is recruited to sites of UV damage (8), we investigated whether UV-induced NONO degradation was dependent on RNF8. To address this question, we performed siRNA-mediated depletion of RNF8 in HeLa cells followed by UV irradiation. RNF8 knockdown inhibited UV-induced reduction of NONO (Figure 3A), suggesting that RNF8 targets NONO for degradation in response to UV-induced DNA damage. Next, we sought to determine whether UV radiation affects the ubiquitination of NONO. For this purpose, HeLa cells were exposed to UV (30 J/m^2^) for the indicated times and levels of immunoprecipitated NONO ubiquitination was determined using Western blotting analysis. As shown in Figure 3B, UV radiation resulted in increased ubiquitination of NONO in a time-dependent manner. The ubiquitination smear observed was derived from NONO itself but not from proteins bound to NONO, since SFPQ which forms a stable complex with NONO in the cell was not detected under our harsh IP condition for the ubiquitination assay.

Next, to examine whether UV light exposure has a similar effect on ubiquitination of other members of DBHS family namely SFPQ and PSPC1, we transfected HeLa cells with plasmid encoding FLAG-NONO, FLAG-SFPQ or FLAG-PSCP1 and irradiated with UV (30 J/m^2^). Strikingly, the *in vivo* ubiquitination assay data revealed that UV-induced DNA damage elicited ubiquitination of neither SFPQ nor PSCP1, suggesting that UV radiation specifically induces NONO ubiquitination (Figure 3C). We further investigated whether RNF8 is involved in UV-induced NONO ubiquitination. To test this hypothesis, we transfected HeLa cells with control or RNF8-specific siRNA and then exposed the cells to UV (30 J/m^2^). We found that the UV-induced ubiquitination of NONO was reduced in RNF8-depleted cells compared to control siRNA-treated cells (Figure 3D). Taken together, these results suggest that RNF8 promotes ubiquitination and subsequent degradation of NONO in response to UV-induced DNA damage.

### Mapping lysine residues of NONO conferring its instability in response to UV-induced DNA damage

To understand the physiological significance of NONO degradation by RNF8 in UV-DDR, we attempted to generate a more stable derivative of NONO against UV-induced DNA damage. Since two lysine residues at positions 198 and 371 of NONO were identified as sites of ubiquitin conjugation (Supplementary Figure S1B), we first substituted these lysine residues with arginine and monitored NONO protein stability following UV exposure. The NONO K198, 371R mutant was not significantly stabilized compared to wild-type NONO (data not shown), suggesting that other lysine residues can be used as ubiquitination sites in the mutant protein.

**Figure 4.**
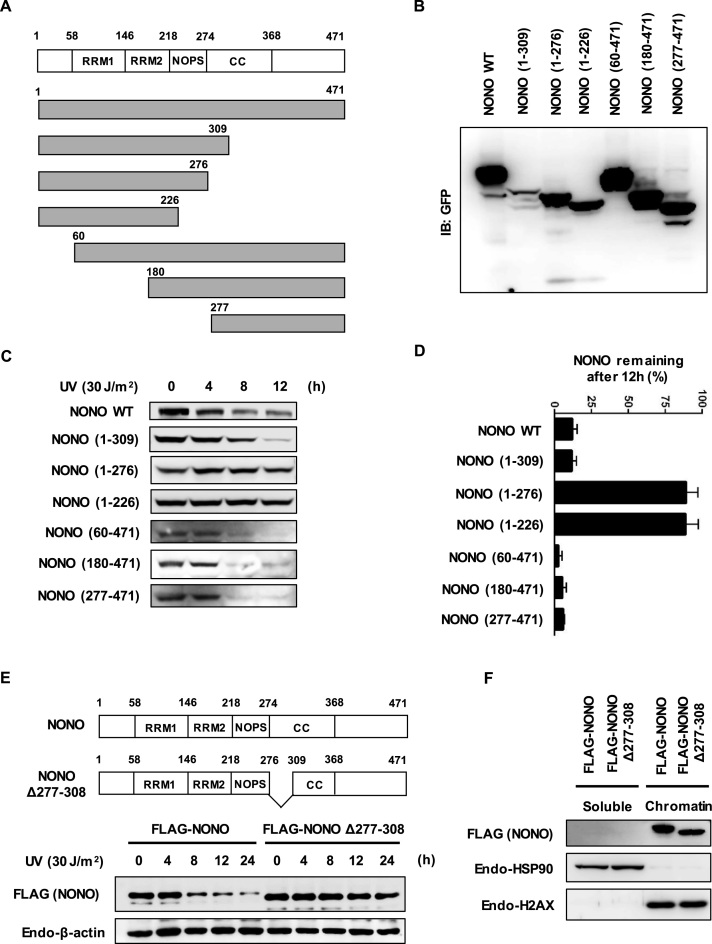
Mapping the region of NONO which confers instability in response to UV-induced DNA damage. (**A**) Schematic representation of NONO structure (RRM1 & 2: RNA recognition motifs 1 and 2, NOPS: NonA/paraspeckle domain, and CC: Coiled-coil domain) and strategy for mutagenesis. (**B**) Expression analysis of NONO deletion mutants. HEK-293 cells were transfected with pEGFP-C1-NONO and a series of pEF/myc/nuc/GFP-NONO mutant constructs. Twenty hours after transfection, cells were harvested and processed for Western blotting. (**C**) Analysis of protein stability for NONO deletion mutants after UV. HeLa cells were transfected with plasmids encoding GFP-tagged NONO and GFP-NLS-tagged NONO deletion mutants, and treated with UV (30 J/m^2^) for the indicated time points. (**D**) Graphical representation of (**C**) illustrates GFP-tagged NONO and GFP-NLS-tagged NONO deletion mutants’ protein stability after UV. Error bars represent standard deviations from three independent experiments. (**E**) Generation of stable NONO deletion mutant based on the protein degradation information summarized in (C and D). Amino acid residues from 277 to 308 were deleted to generate a stable NONO deletion mutant. (**F**) Stable NONO deletion mutant localizes to the chromatin-enriched fraction. HeLa cells expressing FLAG-NONO and FLAG-NONO Δ277-308 were assayed for chromatin fractionation, and separated into soluble and chromatin-enriched fractions. The collected fractions were analyzed by Western blotting using an anti-H2AX antibody as a marker for the chromatin-enriched fraction and an anti-HSP90 antibody as a marker for the soluble fraction.

To identify the major sites of ubiquitination between the 27 lysine residues dispersed throughout the length of NONO, we first chose to determine the region required for UV-induced NONO degradation. We designed a set of GFP-nuclear localization signal (NLS)-tagged NONO deletion mutants as shown in Figure 4A, verified their expression (Figure 4B), and then determined the protein stability of NONO wild-type and deletion mutants in response to UV ray in HeLa cells. NONO (1–226) and NONO (1–276) mutants were significantly more stable than wild-type NONO or NONO (1–309), NONO (60–471), NONO (180–471) and NONO (277–471) deletion mutants; the latter group of mutants were efficiently degraded following UV exposure (Figure 4C and D). These data suggest that the region between residues 277 and 308 of NONO is required for its UV-induced degradation. To further confirm our finding, we generated a NONO Δ277-308 mutant by removing a stretch between residues 277 and 308, and compared the degradation of this internally deleted mutant with wild-type NONO in response to UV irradiation. We observed that the NONO Δ277–308 mutant was much more stable than wild-type NONO following UV exposure (Figure 4E), indicating involvement of the deleted segment in UV-induced degradation of NONO. The deletion of these 32 internal amino acid residues might cause a localization defect of NONO to impair its UV-induced degradation. To exclude this possibility, we expressed FLAG-tagged NONO or FLAG-tagged NONO Δ277-308 mutant in HeLa cells and fractionated cell extracts into soluble proteins and chromatin-bound proteins for subsequent immunoblotting analysis. Wild-type NONO was mainly distributed in chromatin-enriched fractions as reported previously (35). The NONO Δ277–308 mutant was also exclusively distributed to chromatin-enriched fractions (Figure 4F), suggesting that enhanced stability of NONO Δ277-308 mutant does not result from mislocalization of the mutant protein.

**Figure 5.**
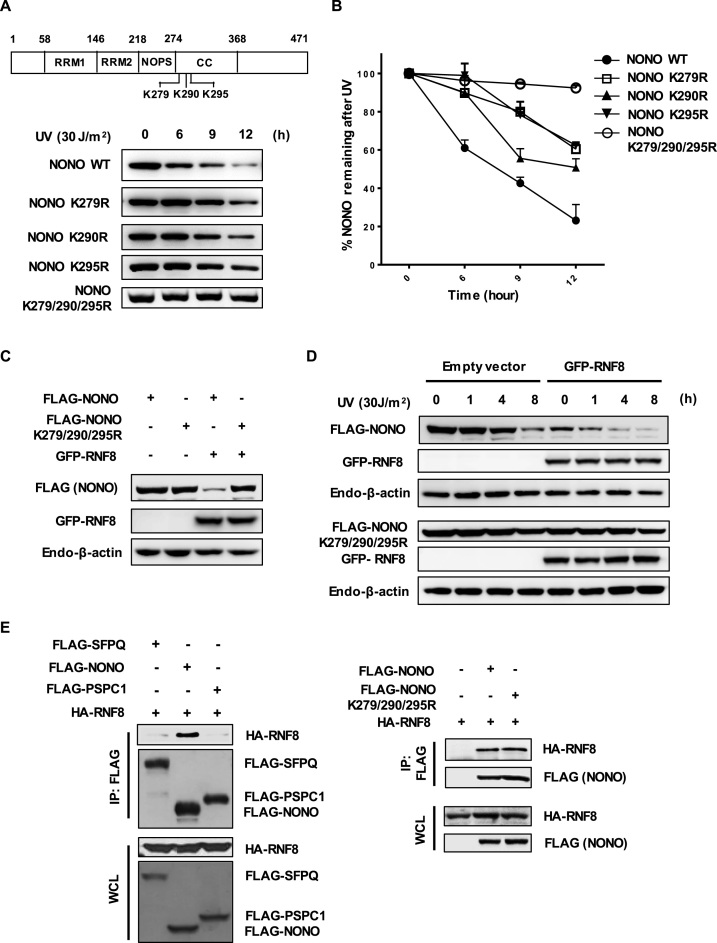
The NONO lysine residues 279, 290 and 295 are crucial for RNF8-dependent UV-induced NONO degradation. (**A**) Determination of protein stability for NONO lysine mutants in response to UV DNA damage. HeLa cells transfected with FLAG-tagged NONO and FLAG-tagged NONO lysine mutants were treated with UV (30 J/m^2^) for the indicated time points. Whole cell lysates were analyzed by Western blotting using an anti-FLAG antibody. (**B**) Graphical representation of (A) shows the protein stability of FLAG-tagged NONO and FLAG-tagged NONO lysine mutants after UV irradiation. Error bars represent standard deviations from three independent experiments. (**C**) The stable triple lysine mutant NONO is resistant to RNF8-mediated proteolysis. HeLa cells were transfected with FLAG-NONO or FLAG-NONO K279/290/295R in the presence or absence of GFP-RNF8. Cell lysates were analyzed by Western blotting using the indicated antibodies. (**D**) Ectopically expressed RNF8 fail to degrade the triple lysine mutant NONO after UV exposure. HeLa cells transfected with an empty vector or GFP-RNF8 with FLAG-NONO or FLAG-NONO K279/290/295R were irradiated with UV (30 J/m^2^) for the designated time points. The cell lysates were probed with the indicated antibodies for Western blotting. (**E**) RNF8 interacts with NONO but not with SFPQ or PSPC1. HeLa cells were co-transfected with HA-RNF8 plus FLAG-SFPQ, FLAG-NONO or FLAG-PSPC1. Cell lysates were immunoprecipitated with an FLAG antibody (left panel). The triple lysine-to-arginine mutation of NONO (K279R, K290R and K295R) does not interfere the binding of NONO with RNF8. HeLa cells were co-transfected with HA-RNF8 plus an empty vector or FLAG-NONO or FLAG-NONO K279/290/295R. Cells were treated with 25 μM MG132 for 4 h. Cell lysates from transfected cells were used for immunoprecipitation analysis with an FLAG antibody (right panel). Both whole cell lysates and immunoprecipitated complexes were visualized by Western blotting with the indicated antibodies.

Three lysine residues (K279, K290 and K295) reside within the deleted segment of the NONO Δ277–308 mutant. To investigate the effect of these residues on UV-induced NONO degradation, we generated three single lysine-to-arginine mutants (K279R, K290R and K295R) and a triple mutant (with lysines 279, 290 and 295 mutated to arginine [K279/290/295R]), and compared the stability of these proteins to wild-type NONO following UV exposure. Each single lysine mutant was more stable than wild-type NONO after UV radiation with K279R and K295R mutations showing greater stabilizing effect, indicating that these lysine residues contribute to NONO instability (Figure 5A and B). The most dramatic increase in NONO stabilization following UV exposure was consistently observed with the triple mutant. Next, we examined whether the triple lysine-to-arginine mutation of NONO confers resistance to RNF8. As shown in Figure 5C, the triple mutant was not appreciably degraded by RNF8. Furthermore, NONO K279/290/295R survived the combination of RNF8 overexpression and UV exposure (Figure 5D).

We hypothesize that the K279/290/295R residues could confer NONO stability against RNF8 because they are either: i) the major sites of ubiquitination or ii) important for the binding of RNF8. To distinguish between these two possibilities, we examined the interaction of HA-RNF8 with FLAG-tagged wild-type and the triple mutant NONO, SFPQ or PSPC1 by co-immunoprecipitation analysis. As shown in Figure 5E and Supplementary Figure S10, HA-RNF8 showed a robust interaction with FLAG-NONO, but not with FLAG-SFPQ or with FLAG-PSPC1, explaining why RNF8 directs ubiquitination of NONO specifically among the DBHS family members. Interaction studies with RNF8 deletion mutants revealed that the N-terminal part of RNF8 including the FHA domain is required for its interaction with NONO (Supplementary Figure S10C). The triple lysine-to-arginine mutation of NONO did not block its interaction with RNF8 (Figure 5E), suggesting that the three lysine residues are not critical for the binding of NONO to RNF8. To investigate whether the three lysine residues are indeed involved in ubiquitination, we mapped ubiquitination sites on NONO by mass spectrometry following overexpression of FLAG-NONO and HA-RNF8 (the data has been deposited at ProteomeXchange database, www.proteomexchange.org, identifier: PXD006156). We identified 11 lysine residues as ubiquitination sites on NONO, including K279 and K295 (Supplementary Table S1 and Supplementary Figure S11), consistent with the idea that these lysines are important ubiquitination sites. We further confirm this result using *in vivo* ubiquitination assay and showed that the ubiquitination level of NONO was significantly decreased in triple lysine NONO mutant compared to the wild-type NONO in cells expressing exogenous RNF8 (Supplementary Figure S12). Taken together, these results indicate that lysines 279, 290 and 295 of NONO are critical for UV-induced NONO degradation and strengthens our conclusion that UV-induced NONO degradation is mediated by RNF8.

### RNF8-mediated degradation of NONO is required for S phase progression by terminating ATR-CHK1 checkpoint signaling in UV-DDR

NONO was recently found to aid TOPBP1 loading onto damaged chromatin to activate ATR following UV exposure (44). Consistent with this notion, our data clearly demonstrated that depletion of NONO significantly decreased the loading of TOPBP1 and ATRIP on the chromatin during UV-DDR (Supplementary Figure S13A). To further elucidate the role of stable NONO on loading of TOPBP1 and ATRIP after UV-exposure, we transfected HeLa cells with FLAG-NONO and FLAG-NONO K279/290/295R and monitored the retention of TOPBP1 and ATRIP on the chromatin. We found that the triple lysine mutant NONO stabilizes TOPBP1 and ATRIP on the chromatin compared to wild type NONO (Supplementary Figure S13B). These finding suggests that stabilization of NONO promotes the retention of TOPBP1 and ATRIP on the chromatin.

**Figure 6.**
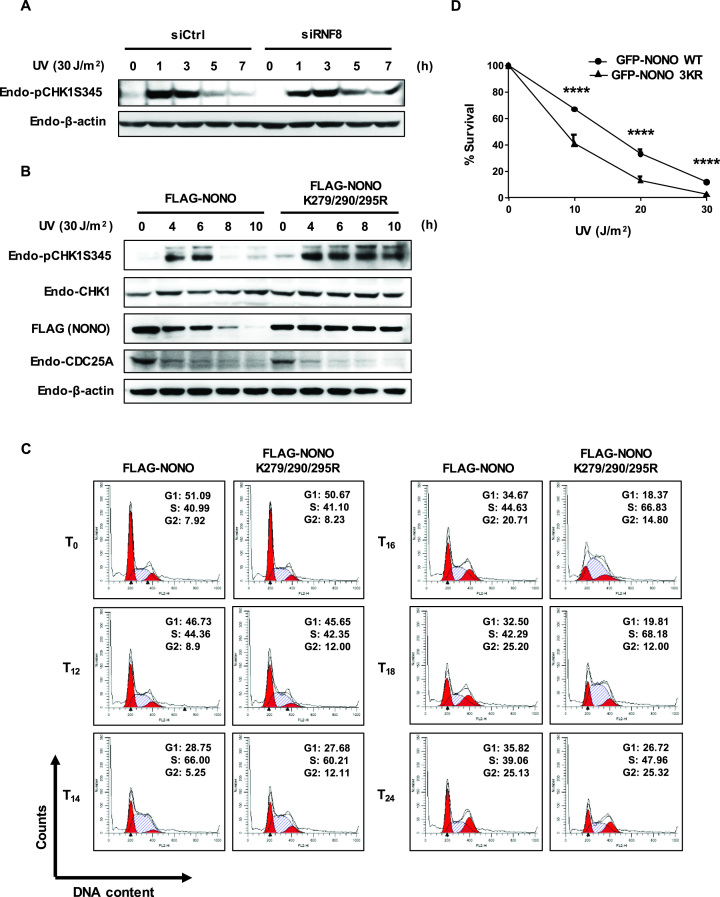
RNF8-mediated degradation of NONO is required for S phase progression by terminating ATR-CHK1 checkpoint signaling in UV-DDR. (**A**) RNF8 depletion causes sustained ATR-CHK1 checkpoint signaling during UV DNA damage. HeLa cells transfected with control siRNA or RNF8 siRNA were exposed to UV (30 J/m^2^) radiation for the designated time points. Cells were lysed and processed for Western blotting analysis. (**B**) Ectopic expression of stable triple lysine mutant NONO leads to prolonged activation of ATR-CHK1 checkpoint signaling. HeLa cells were transfected with FLAG-NONO and FLAG-NONO K279/290/295R and then treated with UV (30 J/m^2^) for the indicated time points. Cell lysates were probed with the indicated antibodies for Western blotting analysis. (**C**) Stabilization of NONO delays S-phase progression after UV DNA damage. HeLa cells transfected with FLAG-NONO and FLAG-NONO K279/290/295R were irradiated with UV (10 J/m^2^) for the indicated time intervals, and DNA content was analyzed by FACS analysis using propidium iodide staining. The cell cycle profiles were quantified and depicted in the top right corner of the figures. (**D**) Clonogenic survival of U2OS-derived stable cell lines expressing the GFP-tagged NONO wild type or the triple lysine (3KR) NONO mutant after UV radiation. Curves show mean ± s.d. of three independent experiments. ****P < 0.0001; *P*-values represent two-way ANOVA results.

Since RNF8 is responsible for UV-induced NONO degradation, we next investigated whether depletion of RNF8 affects ATR by assessing CHK1 phosphorylation at serine 345 following UV exposure. As shown in Figure 6A, RNF8 knockdown resulted in strong phosphorylation of CHK1 even 7 h after UV exposure, indicating sustained ATR-CHK1 checkpoint signaling. This finding is consistent with the notion that UV-induced RNF8-dependent NONO degradation is required for proper checkpoint signaling.

To directly address the relevance of UV-induced NONO degradation in checkpoint response, we expressed wild-type or triple lysine mutant (ie, stable) NONO in HeLa cells and monitored the status of CHK1 phosphorylation following UV exposure by Western blotting. Whereas the phosphorylation of CHK1 was maintained until 6 h after exposure to UV in cells expressing wild-type NONO, NONO K279/290/295R prolonged CHK1 phosphorylation over an extended period of time (Figure 6B and Supplementary Figure S14), indicating that NONO degradation is essential for the termination of checkpoint signaling. Since CDC25A, a downstream target of CHK1, is known to be degraded in response to UV DNA damage (62), we examined CDC25A levels following UV radiation. Although not as dramatic as its effect on CHK1 phosphorylation, NONO-3KR mutant appears slightly more efficient in reducing CDC25A than wild-type NONO, suggesting that CDC25A degradation following UV exposure partially correspond with sustained CHK1 activation in NONO mutant expressing cells.

In UV-DDR, ATR-CHK1 pathway is activated to regulate various phases of the cell cycle, including the S phase (63–67). To investigate the effect of sustained phosphorylation of CHK1 by stabilized NONO on cell cycle progression, cells were transfected with wild-type or the triple lysine mutant (ie, stable) NONO, irradiated with UV (10 J/m^2^), and then cell cycle profiles were analyzed by flow cytometry. Significantly higher fractions of cells expressing NONO K279/290/295R were in S phase than cells expressing wild-type NONO after 16, 18 and 24 h after UV exposure (Figure 6C), indicating that the stable NONO mutant induces prolonged S phase after UV exposure. We next investigated the effect of NONO stabilization in cell survival upon UV exposure. For this, we irradiated U2OS-derived stable cell lines expressing GFP-tagged wild type or the triple lysine mutant NONO with the increasing doses of UV and 7 days later colony formation was assayed. As shown in Figure 6D, cells expressing GFP-tagged triple lysine mutant display increased UV sensitivity compared to cells expressing wild-type NONO, indicating that the stable NONO mutant expression increases UV-induced cell death. Taken together, these findings indicate that RNF8-dependent NONO degradation after UV exposure is essential for S-phase progression and has a protective role against UV-induced cell death by proper termination of ATR-CHK1 checkpoint signaling in UV-DDR.

## DISCUSSION

RNF8 plays a crucial role in DSB-DDR to initiate ubiquitination-dependent signaling, through which downstream DDR factors (eg, 53BP1 and BRCA1) are recruited to sites of DNA damage to repair DSBs by non-homologous end joining or homologous recombination. In UV-DDR, RNF8 is recruited to sites of UV damage leading to the accumulation of a similar set of DDR factors (8). However, the functional significance of RNF8 in UV-DDR is not fully understood. In this study, we provide novel insight into the RNF8-mediated regulation of ATR-CHK1 pathway in UV-DDR. Our data show that following UV-induced DNA damage, RNF8 promotes NONO degradation to switch off signaling through the ATR-CHK1 pathway.

**Figure 7.**
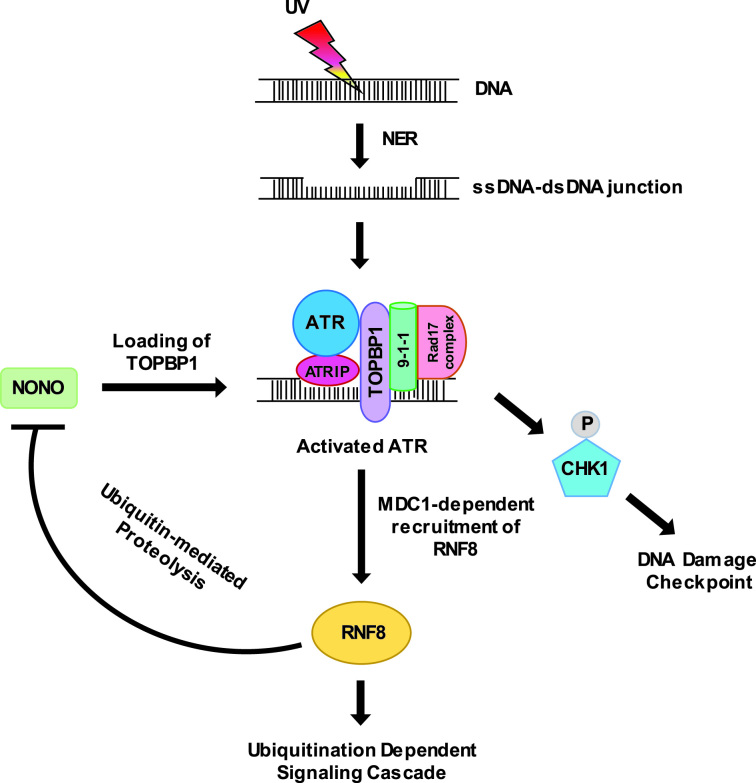
A proposed model for RNF8-mediated negative feedback regulation of ATR-CHK1 checkpoint signaling through NONO degradation in response to UV DNA damage.

UV-induced DNA damage produces stretches of single-stranded DNA (ssDNA) during NER (68). Replication protein A (RPA)-coated ssDNA binds ATR-interacting protein (ATRIP) in complex with ATR (69). Independently, the Rad17 complex, containing four small subunits of RFC (RFC2-RFC5), is recruited to the adjacent double-strand DNA (dsDNA). The Rad17-RFC protein complex helps the loading of the clamp-shaped Rad9-Rad1-Hus1 (9-1-1 complex) to 5′ dsDNA-ssDNA junctions (70,71). Subsequent recruitment of TOPBP1, which interacts with Rad9, ATRIP and ATR, leads to full activation of ATR (72–74). ATR phosphorylates H2AX (75), which recruits MDC1. MDC1 helps with the accumulation of TOPBP1 through direct protein-protein interaction (76), forming a feed-forward loop to promote ATR activation. Recently, it was reported that NONO interacts with TOPBP1 (77) and promotes its chromatin loading to activate ATR (44). RNF8 was shown to be recruited to sites of UV damage by MDC1 in an ATR-dependent fashion (8). Our data show that RNF8 promotes NONO ubiquitination and its proteasome-dependent degradation. Thus, as depicted in Figure 7, ATR directs phosphorylation of CHK1 to activate checkpoint signaling, while at the same time promoting RNF8 recruitment to induce NONO degradation and the subsequent inhibition of TOPBP1-dependent ATR activation. We propose that this negative feedback loop involving RNF8-mediated NONO degradation is critical for turning off ATR-CHK1 checkpoint signaling in UV-DDR.

Recently, it was reported that 10 J/m^2^ of UV radiation did not induce any changes at the mRNA or the protein level of NONO (78). Interestingly, UV-induced microRNA320a targeted NONO mRNA for its downregulation; however, RNA-binding protein HUR inhibited NONO mRNA degradation by interfering with miRNA320a binding to NONO mRNA in response to UV (78). Our findings, however, indicated that UV induced NONO downregulation by protein degradation but not by microRNA-mediated mRNA degradation or translation inhibition. UV radiation lowered NONO protein levels in a dosage-dependent fashion, which was blocked by proteasome inhibition (Figure 2). UV-induced NONO downregulation was also observed in the presence of the translation inhibitor cycloheximide, suggesting that UV induced degradation of pre-existing NONO. Furthermore, local nuclear UV damage resulted in local decrease of NONO protein, which cannot be explained by any process requiring new protein synthesis. In addition, we found that UV induces RNF8-mediated NONO ubiquitination (Figure 3). Together, these data strongly argue for the ubiquitination- and proteasome-dependent degradation of NONO by UV.

We found that three lysine residues (K279, K290 and K295) are critical for UV-induced RNF8-dependent NONO degradation. These residues reside in the N-terminal part of the coiled-coil domain which facilitates dimerization of the DBHS proteins. Recent structural studies revealed that these residues are not involved in the heterodimer interface (79), suggesting that the triple lysine-to-arginine mutation is not likely to affect the dimer integrity or to induce gross conformational changes of NONO. Our mass spectrometric analysis showed K279 and K295 are indeed ubiquitin-conjugation targets. It is currently not clear whether K290 is involved in ubiquitination.

DBHS proteins function as obligatory dimers. The best-characterized one so far is the NONO-SFPQ heterodimer. The so-called DBHS region of approximately 300 amino acids is very similar in structure between DBHS family members. Furthermore, the three lysine residues involved in RNF8-mediated ubiquitination of NONO are conserved in SFPQ. However, our data suggests that NONO is the only member of the DBHS family regulated by UV radiation and RNF8. Thus, it seems likely that each member of DBHS family is separately regulated. Interestingly, recent reports using stable GFP-based reporter cell lines show that SFPQ is required for homologous recombination repair of DSBs (38), while NONO promotes DSB repair by NHEJ (35). Since the DBHS proteins can form six different dimers, systematic investigation is needed to clarify roles of each member in the context of a dimer.

In summary, our study uncovered the function of RNF8 in checkpoint regulation in UV-DDR. Importantly, we found that although NONO is required for ATR-CHK1 signaling pathway, it should be degraded in a RNF8- and proteasome-dependent way to terminate the checkpoint signaling pathway. Given that ATR functions in various DNA repair and checkpoint responses, it remains to be seen if NONO and other DBHS members are involved in these processes.

## DATA AVAILABILITY

The data has been deposited at ProteomeXchange database, www.proteomexchange.org, identifier: PXD006156

## Supplementary Material

Supplementary Data
