# Supplementary material for: RNF8 mediates NONO degradation following UV-induced DNA damage to properly terminate ATR-CHK1 checkpoint signaling
Source: Nucleic Acids Res. 2018 Nov 16;47(2):762–78. doi: 10.1093/nar/gky1166 (PMC6344893; doi:10.1093/nar/gky1166)
Supplement: Supplementary Data [file gky1166_supplemental_files.pdf]

Figure S1

A

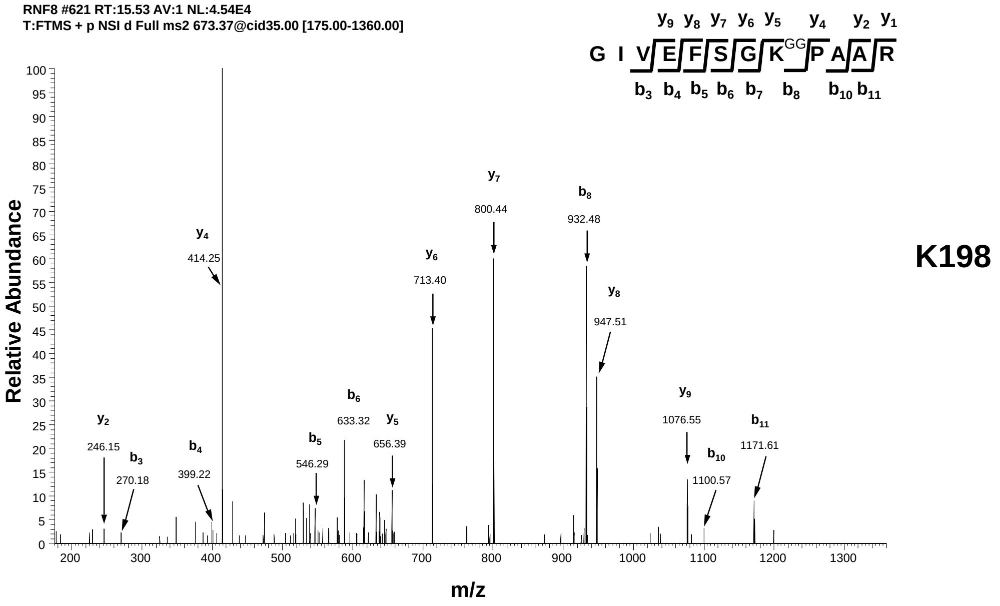

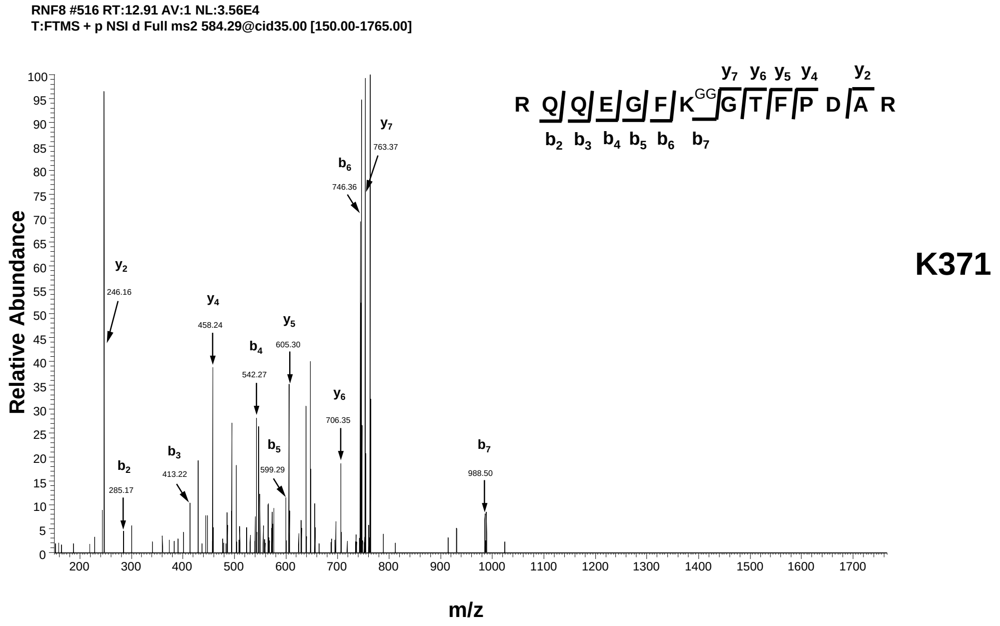

B

Recovered Gly-Gly-NONO peptides

Protein	Ubiquitination site	Peptide Sequence	Mass Deviation (ppm)
NONO	K198	GIVEFSGK^{GG}PAAR	0.94
	K371	RQQEGFK^{GG}GTFPDAR	0.57

Figure S2

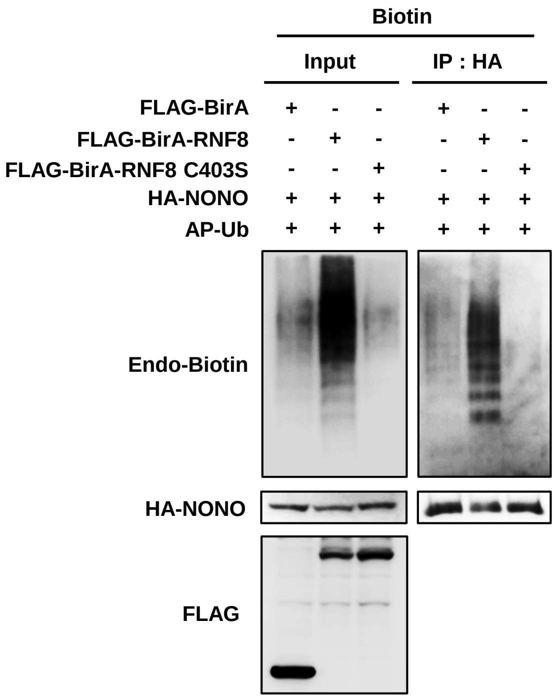

Biotin
Input
IP : HA
FLAG-BirA + - - + - -
FLAG-BirA-RNF8 - + - - + -
FLAG-BirA-RNF8 C403S - - + - - +
HA-NONO + + + + + +
AP-Ub + + + + + +
Endo-Biotin
HA-NONO
FLAG

Figure S3

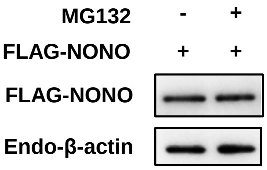

Figure S4

A

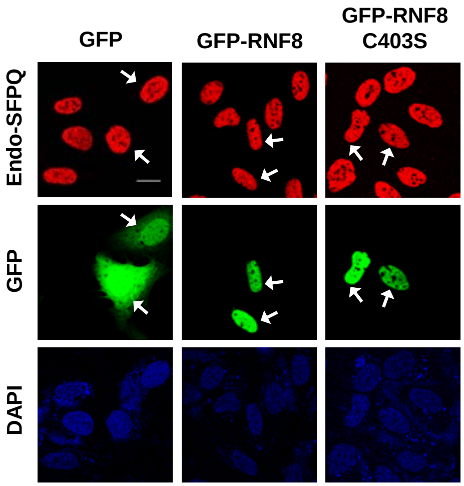
GFP
GFP-RNF8
GFP-RNF8
C403S
Endo-SFPQ
GFP
DAPI

B

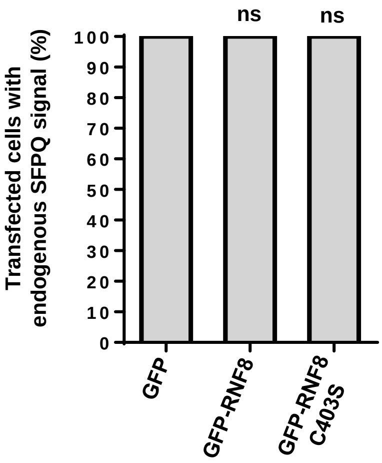
ns
ns
Transfected cells with
endogenous SFPQ signal (%)
100
90
80
70
60
50
40
30
20
10
0
GFP
GFP-RNF8
GFP-RNF8
C403S

Figure S5

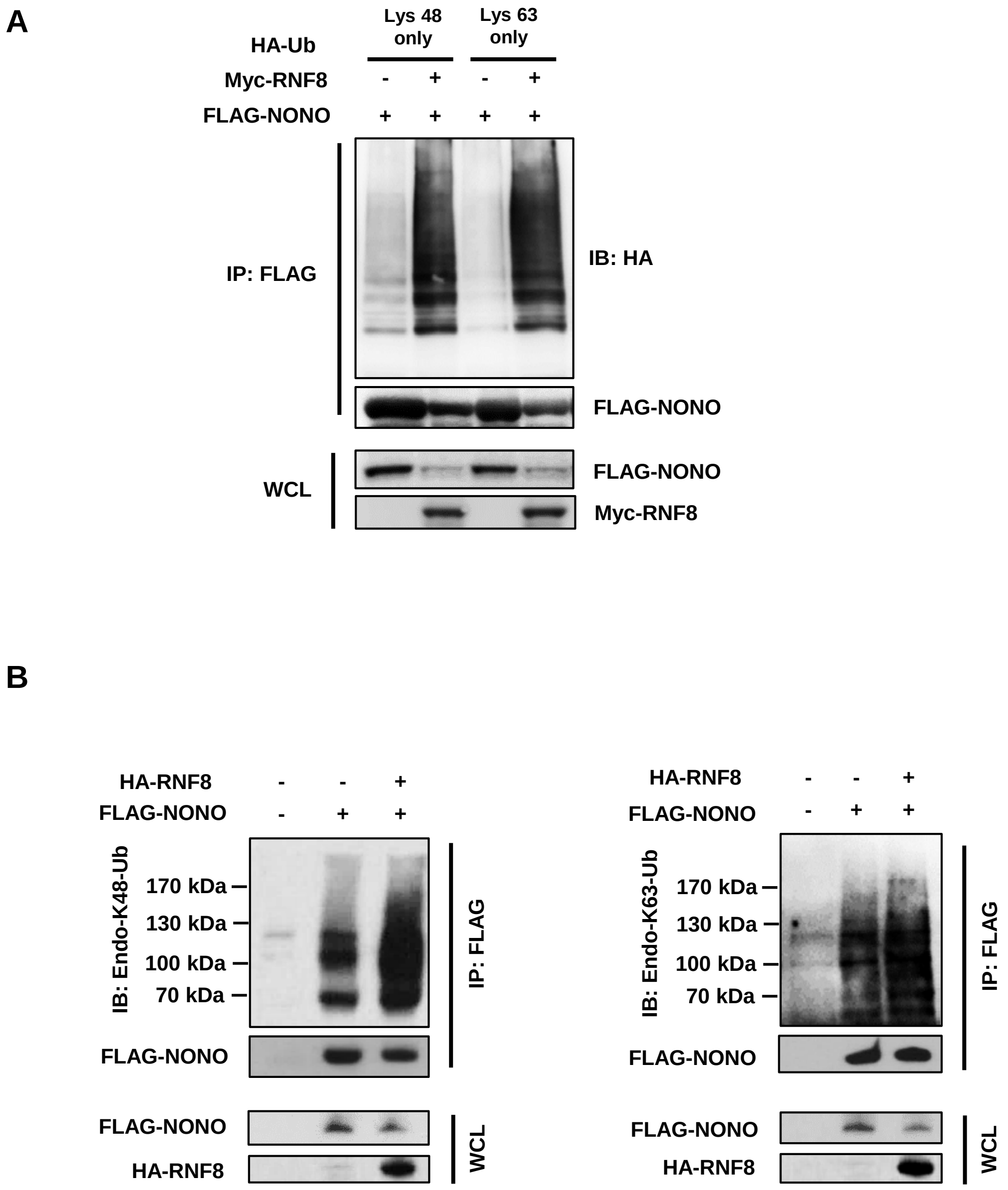
A
HA-Ub
Lys 48 only
Lys 63 only
Myc-RNF8
FLAG-NONO
IP: FLAG
IB: HA
FLAG-NONO
WCL
FLAG-NONO
Myc-RNF8
B
HA-RNF8
FLAG-NONO
IB: Endo-K48-Ub
170 kDa
130 kDa
100 kDa
70 kDa
IP: FLAG
FLAG-NONO
FLAG-NONO
HA-RNF8
WCL
HA-RNF8
FLAG-NONO
IB: Endo-K63-Ub
170 kDa
130 kDa
100 kDa
70 kDa
IP: FLAG
FLAG-NONO
FLAG-NONO
HA-RNF8
WCL

Figure S6

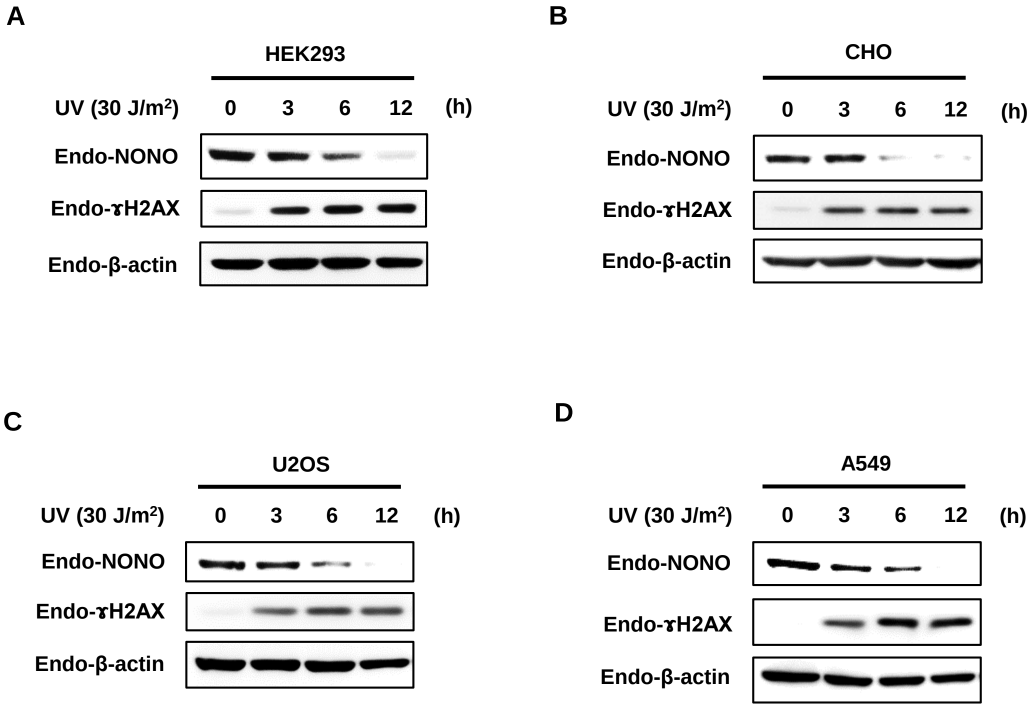

A
HEK293
UV (30 J/m2) 0 3 6 12 (h)
Endo-NONO
Endo-γH2AX
Endo-β-actin
B
CHO
UV (30 J/m2) 0 3 6 12 (h)
Endo-NONO
Endo-γH2AX
Endo-β-actin
C
U2OS
UV (30 J/m2) 0 3 6 12 (h)
Endo-NONO
Endo-γH2AX
Endo-β-actin
D
A549
UV (30 J/m2) 0 3 6 12 (h)
Endo-NONO
Endo-γH2AX
Endo-β-actin

Figure S7

A

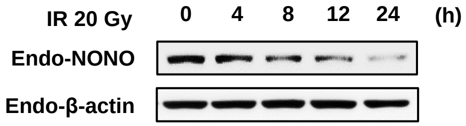

B

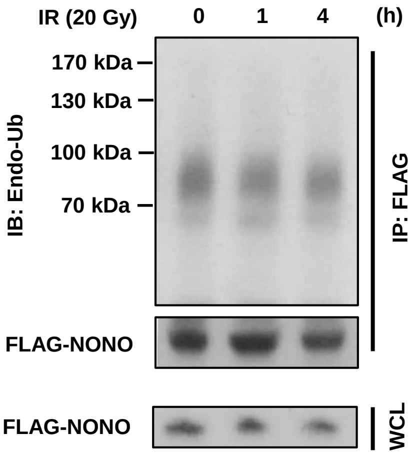

C

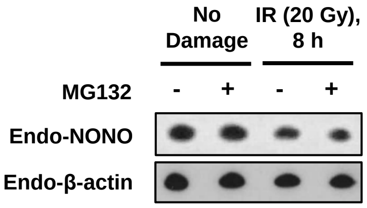

Figure S8

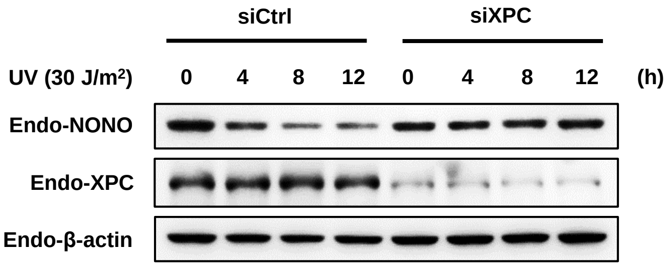
siCtrl
siXPC
UV (30 J/m2)
0
4
8
12
0
4
8
12
(h)
Endo-NONO
Endo-XPC
Endo-β-actin

Figure S9

A

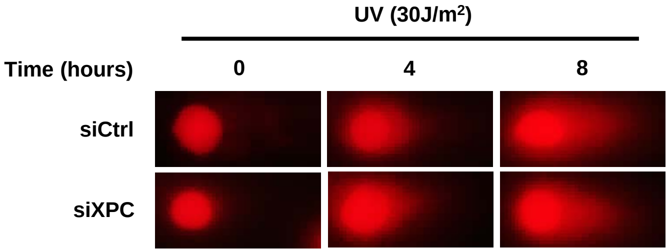

UV (30J/m2)
Time (hours)
0
4
8
siCtrl
siXPC

B

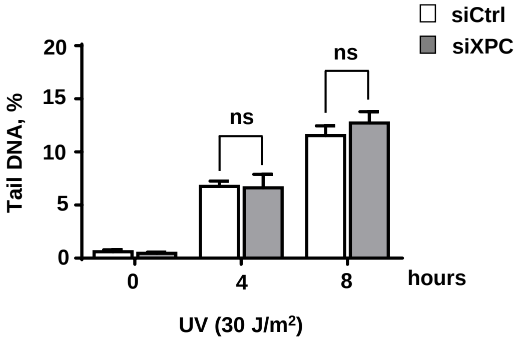

siCtrl
siXPC
20
15
10
5
0
Tail DNA, %
ns
ns
0
4
8
hours
UV (30 J/m2)

Figure S10

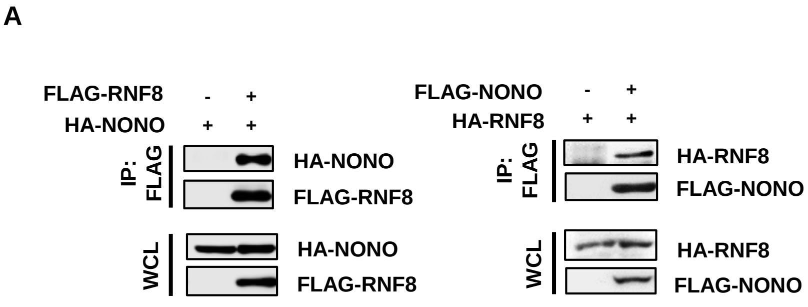
A
FLAG-RNF8 - +
HA-NONO + +
IP: FLAG
HA-NONO
FLAG-RNF8
WCL
HA-NONO
FLAG-RNF8
FLAG-NONO - +
HA-RNF8 + +
IP: FLAG
HA-RNF8
FLAG-NONO
WCL
HA-RNF8
FLAG-NONO

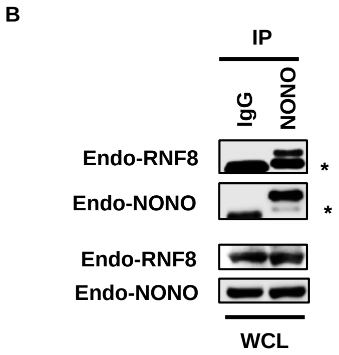
B
IP
IgG
NONO
Endo-RNF8
Endo-NONO
*
*
Endo-RNF8
Endo-NONO
WCL

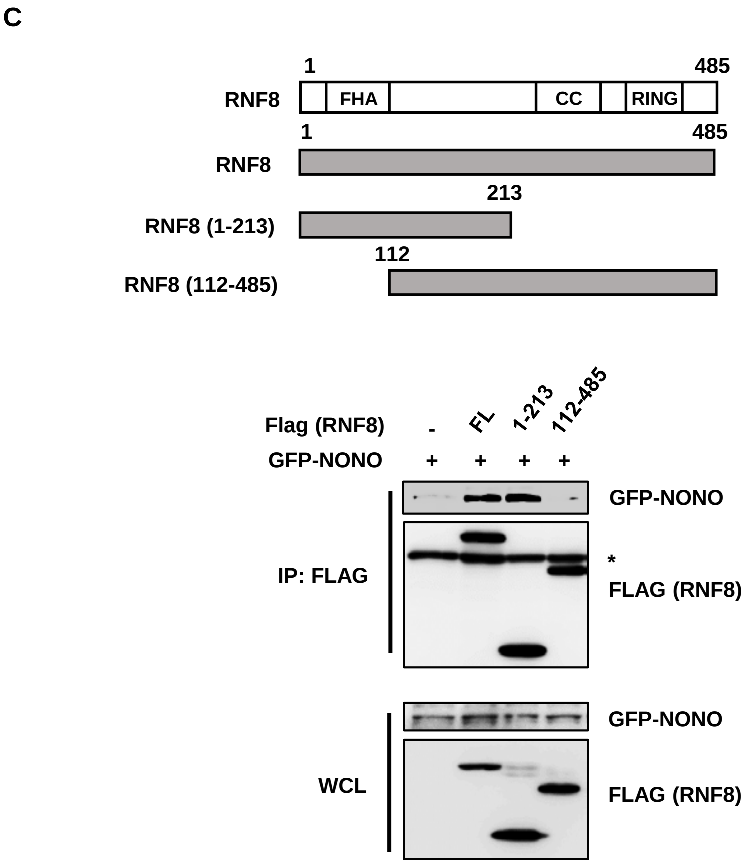
C
1
485
RNF8
FHA
CC
RING
1
485
RNF8
213
RNF8 (1-213)
112
RNF8 (112-485)
Flag (RNF8)
-
FL
1-213
112-485
GFP-NONO + + + +
IP: FLAG
GFP-NONO
*
FLAG (RNF8)
WCL
GFP-NONO
FLAG (RNF8)

Figure S11

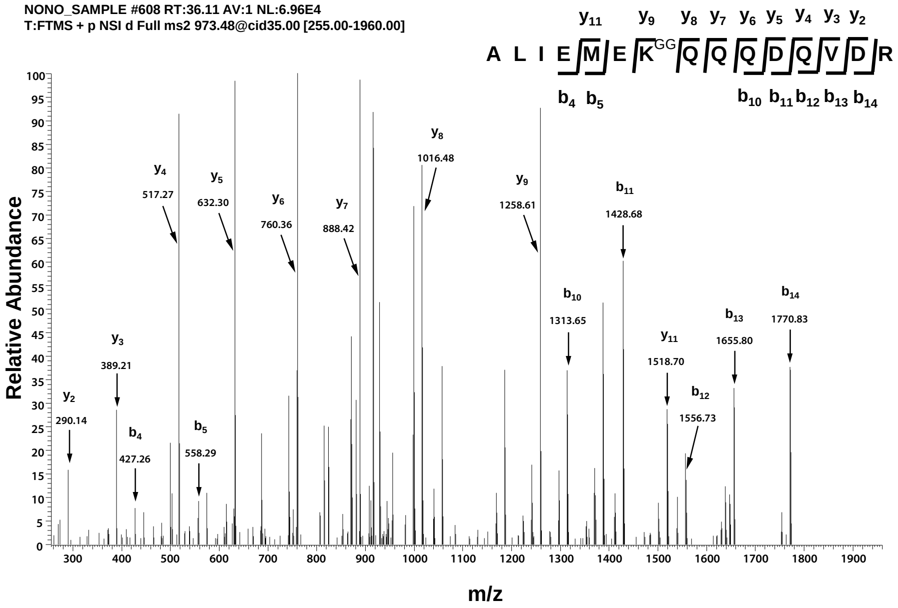

K279

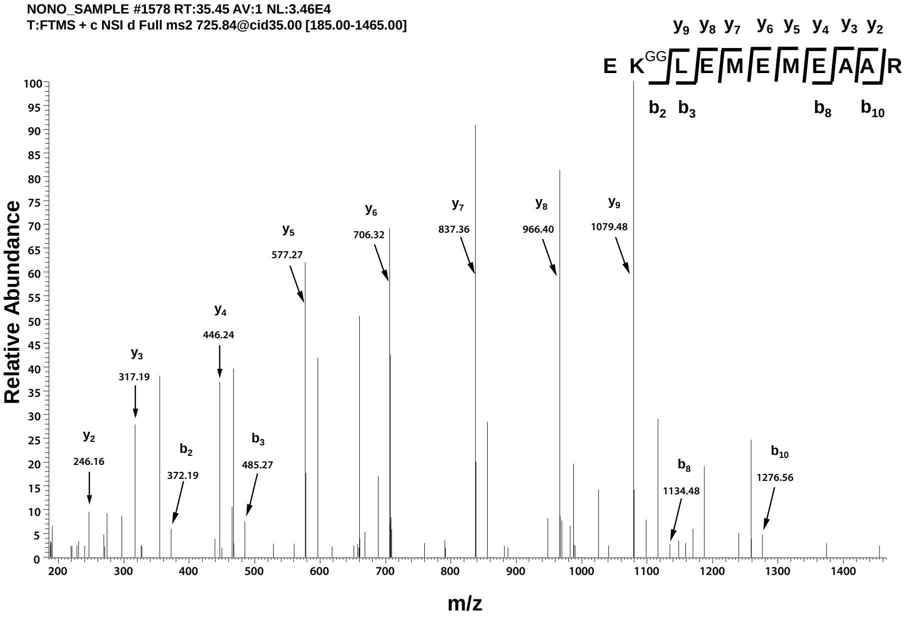

K295

Figure S12

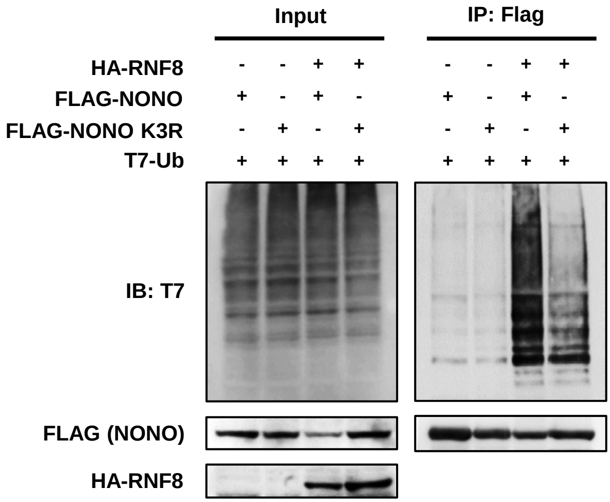
Input
IP: Flag
HA-RNF8
FLAG-NONO
FLAG-NONO K3R
T7-Ub
IB: T7
FLAG (NONO)
HA-RNF8

Figure S13

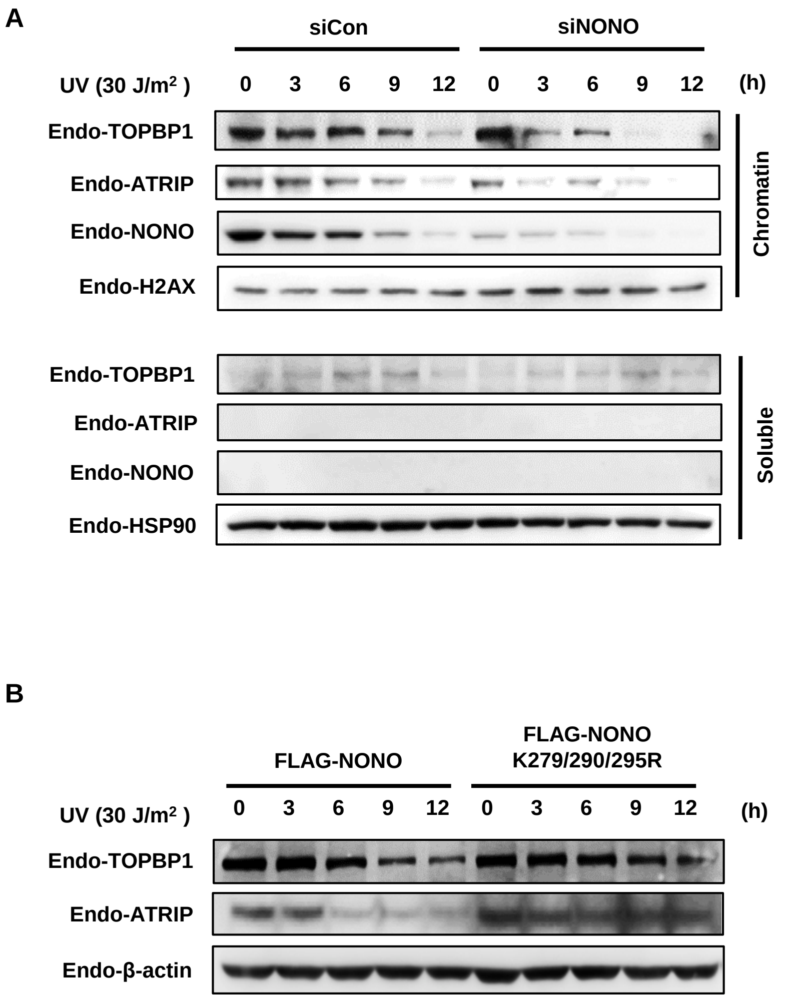
A
siCon
siNONO
UV (30 J/m2)
0 3 6 9 12 0 3 6 9 12 (h)
Endo-TOPBP1
Endo-ATRIP
Endo-NONO
Endo-H2AX
Chromatin
Endo-TOPBP1
Endo-ATRIP
Endo-NONO
Endo-HSP90
Soluble
B
FLAG-NONO
FLAG-NONO
K279/290/295R
UV (30 J/m2)
0 3 6 9 12 0 3 6 9 12 (h)
Endo-TOPBP1
Endo-ATRIP
Endo-β-actin

Figure S14

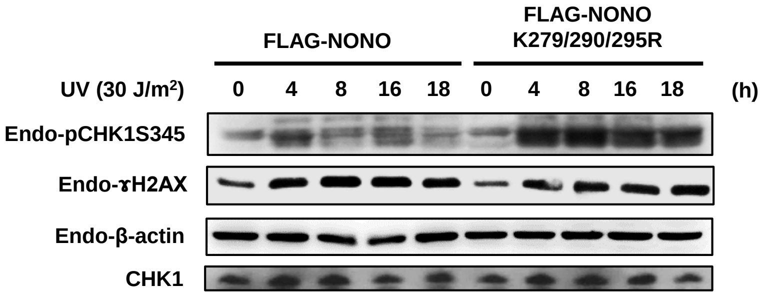
FLAG-NONO
FLAG-NONO K279/290/295R
UV (30 J/m2) 0 4 8 16 18 0 4 8 16 18 (h)
Endo-pCHK1S345
Endo-ɤH2AX
Endo-β-actin
CHK1

Table S1

NONO ubiquitination sites identified in this study

Ubiquitination site	Peptide Sequence	Mass Deviation (ppm)
K11	TFNLEK^{GG}QNHTPR	0.35
K96	LFEK^{GG}YGK	2.8
K99	YGK^{GG}AGEVFIHK	1.68
K109	DK^{GG}GFGFIR	2.77
K126	TLAEIAK^{GG}VELDNMPLR	4.08
K190	GRPSGK^{GG}GIVEFSGKPAAR	0.48
K198	GIVEFSGK^{GG}PAAR	1.54
K239	CSEGSFLLTTFPRPVTVEPMDQLDDEE GLPEK^{GG}LVIK	4.08
K272	WK^{GG}ALIEMEK	2.58
K279	ALIEMEK^{GG}QQQDQVDR	2.24
K295	EK^{GG}LEMEMEAAR	1.87

SUPPLEMENTARY FIGURE LEGENDS

Supplementary Figure S1. Ubiquitin remnant-containing peptides profiling for identification of ubiquitinated proteins. (**A**) Nano-electrospray-MS/MS spectra of ubiquitin remnant NONO peptides. The sequence of ubiquitinated peptides is indicated and the fragment ions (b- and y-ions) are labeled. K^{GG} represents Gly-Gly-modified lysine. (**B**) Summary of ubiquitination sites identified within NONO.

Supplementary Figure S2. Biotinylation of NONO by FLAG-BirA-RNF8. HeLa cells were transfected with AP-Ub, HA-NONO, and FLAG-BirA or FLAG-BirA-RNF8 or FLAG-BirA-RNF8 C403S. Cells were treated with 50 µM biotin for 1 h and processed for ubiquitination assay. Biotin-conjugated NONO was detected by immunoblotting with the indicated antibodies.

Supplementary Figure S3. MG132 treatment has no effect on the level of exogenous NONO protein in cells. HeLa cells were transfected with FLAG NONO and then either treated with 25 µM MG132 for 4 h or not and processed for Western blotting analysis.

Supplementary Figure S4. RNF8 does not degrade SFPQ. (**A**) HeLa cells were transfected with GFP vector or GFP-RNF8 or GFP-RNF8 C403S constructs. Cells were immunostained for SFPQ and visualized using confocal microscopy. Arrows indicate cells expressing GFP or GFP-RNF8 or GFP-RNF8 C403S. Scale bar, 20 µm. (**B**) Quantification of endogenous SFPQ signal in transfected cells was presented in the histogram. Data shown are representative of at least three independent experiments containing 100 cells/sample. Error bars, SD. Student's *t*-test; n.s. not significant.

Supplementary Figure S5. *In vivo* ubiquitination assay was performed in HeLa cells expressing (**A**) FLAG-NONO and HA-ubiquitin mutants containing only one lysine at either position 48 (K48 Ub) or 63 (K63 Ub) with or without Myc-RNF8, or (**B**) FLAG-NONO with or without HA-RNF8. Western blots were probed with K48- and K63-linkage specific polyubiquitin antibodies.

Supplementary Figure S6. UV-induced NONO degradation was examined in various human cell lines.

Supplementary Figure S7. NONO is not regulated by ubiquitin-dependent protein degradation following IR-induced DNA damage. (**A**) HeLa cells were treated with 20 Gy of IR, harvested at the indicated time points, and processed for immunoblotting using the indicated antibodies. (**B**) *In vivo* ubiquitination assay was performed in HeLa cells expressing FLAG-NONO irradiated with 20 Gy IR for the designated time points. Cell lysates were probed with the indicated antibodies for Western blotting analysis. (**C**) The proteasome inhibitor MG132 does not prevent NONO degradation following IR. HeLa cells were irradiated with IR (20 Gy) in the presence of DMSO or 25 µM MG132 for 8 h.

Supplementary Figure S8. XPC depletion prevents NONO degradation after UV radiation. U2OS cells were treated with control siRNA or XPC siRNA and exposed to UV (30 J/m^2) radiation and analyzed after the designated time points. Cells were lysed and processed for Western blotting analysis using the indicated antibodies.

Supplementary Figure S9. (**A**) Neutral comet assay was performed on U2OS control or XPC-

depleted cells following UV (30 J/m^2) exposure at the indicated time points. Representative comet assay images from the experiments are shown. (**B**) Quantification of the comet assay in percent of tail DNA. Error bars, SD. Student's *t*-test; n.s. not significant.

Supplementary Figure S10. RNF8 interacts with NONO. (**A**) Exogenously expressed RNF8 interacts with exogenous NONO. (**B**) Endogenous RNF8 interacts with endogenous NONO. (**C**) RNF8 interacts with NONO through its N-terminal region. HeLa cells were transfected with GFP-NONO together with an empty vector or FLAG-RNF8 or FLAG- RNF8 (1-213) mutant or FLAG-RNF8 (112-485) mutant. Cells were treated with 25 µM MG132 for 4 h and processed for immunoprecipitation assay.

Supplementary Figure S11. Representative MS/MS spectra of peptides demonstrating ubiquitination at lysine 279 and lysine 295 of NONO. The sequence of ubiquitinated peptides is indicated and the fragment ions (b- and y-ions) are labeled. K^{GG} represents Gly-Gly-modified lysine.

Supplementary Figure S12. RNF8-dependent NONO ubiquitination was evaluated in HeLa cells expressing T7-Ub with either the wild-type NONO or the triple lysine (K3R) NONO mutant.

Supplementary Figure S13. (A) Depletion of NONO affects the chromatin loading of TOPBP1 and ATRIP after UV. HeLa cells were treated with either control siRNA or NONO siRNA and exposed to UV (30 J/m^2) radiation for the designated time points. Cells were lysed and separated into soluble or chromatin-enriched fractions as described in Materials and Methods and processed for Western blotting analysis. (**B**) Ectopic expression of the triple lysine (K3R) mutant NONO stabilizes TOPBP1 and ATRIP on the chromatin after UV irradiation. HeLa cells were transfected with FLAG-NONO and FLAG-NONO K279/290/295R and cell lysates were prepared at the indicated time points post UV (30 J/m^2) irradiation. Cell lysates were probed with the indicated antibodies for Western blotting analysis.

Supplementary Figure S14. NONO stabilization prolongs activation of ATR-CHK1 checkpoint signaling. HeLa cells were transfected with FLAG-NONO or FLAG-NONO K279/290/295R and cell lysates were prepared at the indicated time points post UV (30 J/m^2) radiation. Cell lysates were probed with the indicated antibodies for Western blotting analysis.

Supplementary Table S1. Identification of NONO ubiquitination sites.
